# Design and synthesis of new coumarin-based fluorescent chemosensors for the dual detection of Hg^2+^ and Cu^2+^ in aqueous and biological samples

**DOI:** 10.1039/d5ra05643h

**Published:** 2025-11-17

**Authors:** Wahaj Raed Abbas, Mohammed Abed Kadhim

**Affiliations:** a Nanomaterial Research Center, University of Anbar Iraq wah19s3005@uoanbar.edu.iq; b Department of Chemistry, College of Education for Pure Sciences, University of Anbar Iraq

## Abstract

A novel series of coumarin-based Schiff base probes (1a–1d) were designed and synthesized for the highly selective and sensitive dual detection of Hg^2+^ and Cu^2+^ ions in aqueous buffer solutions and human blood serum samples, making them valuable tools in biomedical and environmental applications. These probes exhibited distinct optical responses, a fluorescence “turn-on” mechanism for Hg^2+^*via* chelation-enhanced fluorescence (CHEF) and a quenching effect for Cu^2+^ due to paramagnetic interactions. Probe 1d demonstrated exceptional sensitivity, with detection limits of 36.75 nM for Hg^2+^ and 33 nM for Cu^2+^, along with rapid response times (<35 seconds). Selectivity was confirmed through UV-vis and fluorescence spectroscopy measurements, with minimal interference from other ions. Notably, the probes enabled naked-eye detection through visible color changes (green to yellow for Hg^2+^). Reversibility studies using EDTA highlighted their reusability. Practical applicability was validated by successful Hg^2+^/Cu^2+^ quantification in spiked blood serum samples, achieving recoveries 90–99.8% and 91–99.9% in absorption and fluorescence spectra, respectively. The probe complexes with Hg^2+^/Cu^2+^ could detect S^2−^. These findings underscore the potential of coumarin-based probes as efficient, cost-effective tools for real-time environmental monitoring and clinical diagnostics.

## Abstract 2

New coumarin-based probes (1a–1d) enable highly selective, sensitive, and rapid dual detection of Hg^2+^ and Cu^2+^ in water and blood serum, useful for environmental and biomedical applications.

## Graphical abstract

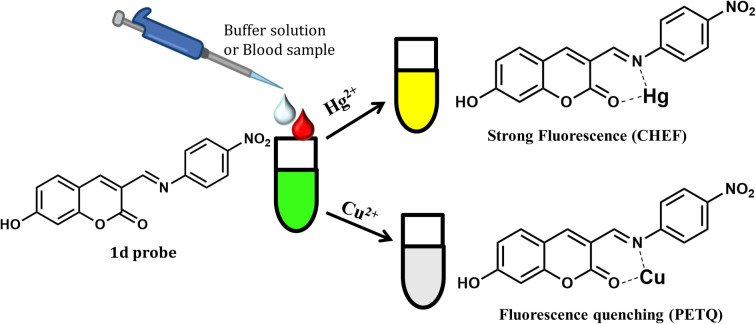

## 1 Introduction

Coumarins are a diverse family of secondary metabolites found in various plant species (more than 1300 coumarins have been identified from natural sources, especially green plants) as well as in fungi and microorganisms.^1,2^ Coumarin is one of the heterocyclic organic compounds that possesses a wide range of biological activities.^3–5^ These heterocyclic compounds (benzopyran-2-one core) have remarkable biological activities, such as anticoagulant, antimicrobial, and anticancer properties.^6–8^ Coumarin derivatives have emerged as highly effective fluorophores and chromophores in chemosensor design due to their excellent photophysical properties, including high quantum yields, visible-light excitation, and significant Stokes shifts.^9–11^ Their structural versatility at the 3-, 4-, or 7-positions of the benzopyrone cores allows for easy modification to enhance their selectivity toward specific metal ions.^12,13^ Heavy metal ions, such as mercury (Hg^2+^) and copper (Cu^2+^), pose significant risks to human health and the environment due to their toxicity, even at trace levels. Mercury exposure can lead to severe neurological and kidney damage, while excessive copper accumulation is associated with neurodegenerative disorders, such as Alzheimer's and Wilson's disease. Consequently, the development of sensitive and selective chemosensors for detecting these metal ions is crucial for environmental monitoring and biomedical diagnostics.^14–17^ In recent years, coumarin-based chemosensors have been engineered to exhibit dual-function detection capabilities, simultaneously identifying Hg^2+^ and Cu^2+^ ions through distinct optical responses. Different techniques are used by coumarin-based dual-detection chemosensors to detect Hg^2+^ and Cu^2+^ ions, allowing for the selective identification of both ions. The major detection mechanisms for Hg^2+^ ions are chelation-enhanced fluorescence (CHEF) and photoinduced electron transfer (PET), while mechanisms for Cu^2+^ detection include chelation-enhanced quenching (CHEQ) and photoinduced electron transfer (PET) quenching.^18,19^ These dual-functional probes offer advantages such as high sensitivity, real-time monitoring, and the ability to discriminate between multiple analytes in complex biological and environmental samples.^20–22^ In this paper, the design and synthesis of new derivatives of coumarin-based chemosensors as dual detection tools for Hg^2+^ and Cu^2+^ metal ions are explored. The probes show significant change in absorbance and fluorescence intensity with a low LOD, while their detection ranges are higher than those of other published probes. Thus, they are applicable in buffer solutions and real samples, like the human blood serum, where they demonstrate acceptable results when used as a kit for biological analysis.

## 2 Experimental

### 2.1 Chemicals

2,4-Hydroxybenzaldehyde and ethyl acetoacetate were procured from Aldrich, Germany, while aniline, *p*-chloroaniline, *p*-methoxy aniline, and *p*-nitroaniline were obtained from BDH. The chemicals were used as received.

### 2.2 Synthesis

**Scheme 1:**
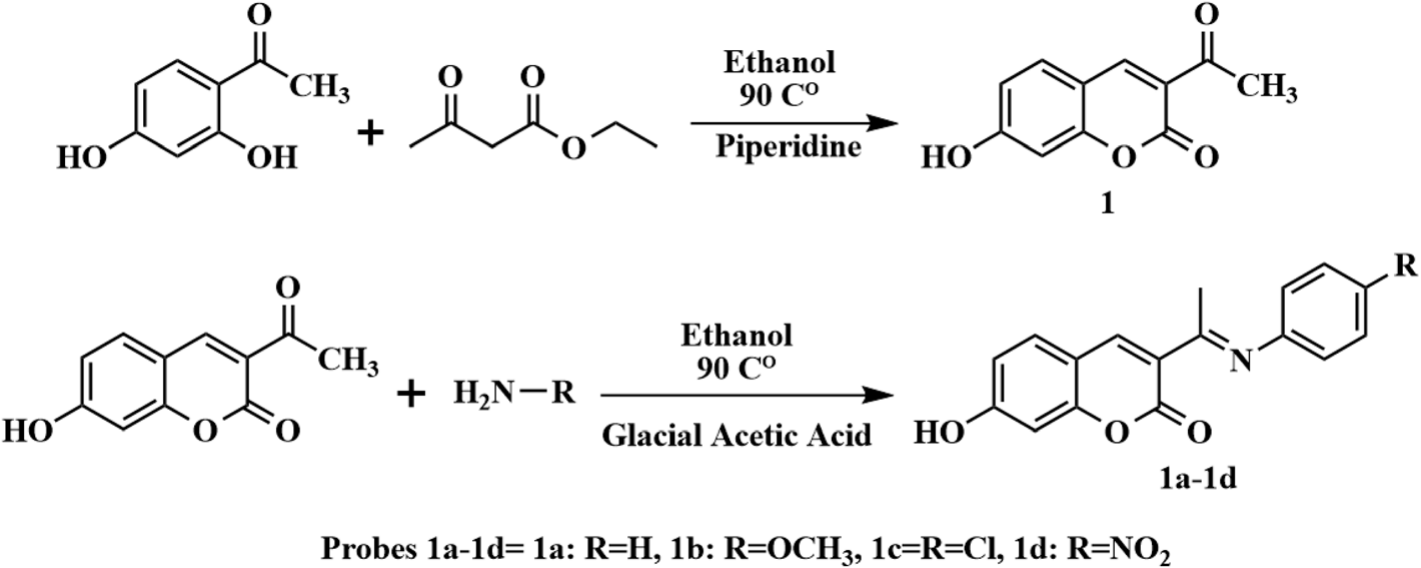
The synthesis route toward intermediates and target compounds 1a–1d.

The synthesis route toward intermediates and target compounds is illustrated in Scheme 1. Compound 1 was synthesized following a procedure mentioned in our previous report.^23^

Target compounds 1a–1d were synthesized according to the method described in our previous work.^24^ The following paragraph describes the 1a synthesis method, and all compounds (1a–1d) were prepared using the same method.

Aniline (0.21 mL, 2.4 mmol) and 3-acetyl-7-hydroxy-2*H*-chromine-2-one 1 (0.5 g, 2.4 mmol) were mixed in 30 mL of pure ethanol with certain amounts of glacial acetic acid droplets acting as a catalyst. The mixture was then left to reflux for 4 hours. Following the evaporation of the excess ethanol, the crude product was dried and separated from the ethanol by recrystallization. A summary of the analytical data for this series of substances can be found below.

#### 2.2.1 1a: 7-Hydroxy-3-(1-(phenylimino)ethyl)-2*H*-chromen-2-one

Yield 80%, M.P 149–151 °C, Chem. Form.: C_17_H_13_NO_3_, M. wt 279.30. FT-IR: *υ*_max_ (cm^−1^): 3480 (OH), 3059 (C–H aromatic), 2926 (C–H aliphatic), 1710 (C=O lactone ring), 1668 (C=N), 1595–1455 (C=C aromatic), 1289 (C–N). ^1^H-NMR δ (ppm, DMSO-d_6_): 11.02 (s, OH), 8.61 (s, H4), 7.78 (d, H6), 6.98 (d, H14 and H15), 6.76 (d, H7), 6.84 (t, H18), 6.75 (s, H9), 6.69 (d, H16, H17), 2.44 (s, H20). ^13^C-NMR δ (ppm): 194.9 (C2), 164.1 (C11), 159.4 (C8), 156.2 (C10), 147.3 (C4), 142.1 (C13), 132.5 (C3), 120.5 (C6), 119.3 (C14, C15), 117.7 (C16, C17), 114.7 (C7), 112.8 (C5), 104.2 (C8), 101.7 (C9), 31.3 (C19). Anal. Found for C_17_H_13_NO_3_ (%): C, 73.11; H, 4.69; N, 5.02; O, 17.

#### 2.2.2 1b: 7-Hydroxy-3-(1-((4-methoxyphenyl)imino)ethyl)-2*H*-chromen-2-one

Yield 81%, M.P: 152–154 °C, Chem. Form.: C_18_H_15_NO_4_, M. wt 309.32. FT-IR: *υ*_max_ (cm^−1^): 3420 (OH), 3080 (C–H aromatic), 2944 (C–H aliphatic), 1715 (C=O lactone ring), 1604 (C=N), 1564–1446 (C=C aromatic), 1241 (C–N), 1194, 1108 (C–O). ^1^H-NMR δ (ppm, DMSO-d6): 11.13 (s, OH), 8.59 (s, H4), 7.80 (d, H6), 6.96 (d, H14 and H15), 6.89 (d, H7), 6.83 (d, H16 and H17), 6.76 (s, H9), 3.62 (s, H19), 2.48 (s, H20). ^13^C-NMR δ (ppm): 194.9 (C2), 164.8 (C11), 159.7 (C8), 157.8 (C10), 151.9 (C18), 148.1 (C4), 142.5 (C13), 132.9 (C3), 122.5 (C14, C15), 119.6 (C6), 116.3 (C7), 114.9 (C16, C17), 111.7 (C5), 102.4 (C9), 56.2 (C19), 31.0 (C20). Anal. Found for C_18_H_15_NO_4_ (%): C, 69.89; H, 4.89; N, 4.53; O, 20.69.

#### 2.2.3 1c: 3-(1-((4-Chlorophenyl)imino)ethyl)-7-hydroxy-2*H*-chromen-2-one

Yield 79%, M.P 157–159 °C, Chem. Form.: C_17_H_12_ClNO_3_, M. wt 313.74. FT-IR: *υ*_max_ (cm^−1^): 3478 (OH), 3058 (C–H aromatic), 2988 (C–H aliphatic), 1715 (C=O lactone ring), 1667 (C=N), 1594–1444 (C=C aromatic), 1289 (C–N). ^1^H-NMR δ (ppm, DMSO-d6): 11.06 (s, OH), 8.58 (s, H4), 7.76 (d, H6), 6.96 (d, H14, H15), 6.86 (d, H16 and H17), 6.83(d, H7), 6.75 (s, H9), 2.45 (s, H19). ^13^C-NMR δ (ppm): 195.1 (C2), 164 (C11), 159.6 (C8), 157.6 (C10), 1478.1 (C4), 142.2 (C13), 132.8 (C3), 130.0 (C16, C17), 124.1 (C18), 120.0 (C6), 119.9 (C14, C15), 114.8 (C7), 111.2 (C5), 102.4 (C9), 31.1 (C19). Anal. Found for C_17_H_12_ClNO_3_ (%): C, 65.08; H, 3.86; Cl, 11.30; N, 4.46; O, 15.30.

#### 2.2.4 1d: 7-Hydroxy-3-(1-((4-nitrophenyl)imino)ethyl)-2*H*-chromen-2-one

Yield 74%, M.P 161–163 °C, Chem. Form.: C_17_H_12_N_2_O_5_, M. wt 324.29. FT-IR: *υ*_max_ (cm^−1^): 3470 (OH), 3061 (C–H aromatic), 2913 (C–H aliphatic), 1719 (C=O lactone ring), 1604 (C=N), 1550–1447 (C=C aromatic), 1500 (N=O), 1291 (C–N). ^1^H-NMR δ (ppm, DMSO-d6): 11.19 (s, OH), 8.58 (s, H4), 7.78 (d, H6), 6.98 (d, H14 and H15), 6.91(d, H16 and H17), 6.84 (d, H7), 6.74 (s, H9), 2.45 (s, H19). ^13^C-NMR δ (ppm): 194.9 (C2), 164.9 (C11), 159.8 (C8), 157.6 (C10), 148.0 (C4), 141.2 (C13), 135.9 (C18), 132.9 (C3), 132.5 (C16, C17), 120.0 (C6), 119.9 (C14, C15), 114.8 (C7), 112.2 (C5), 102.4 (C9), 30.0 (C19). Anal. Found for C_17_H_12_N_2_O_5_ (%): C, 62.96; H, 3.73; N, 8.64; O, 24.67.

### 2.3 Photophysical studies

#### 2.3.1 The absorption and fluorescence properties

The absorption and fluorescence spectral studies of compounds 1a–1d were performed at room temperature in a pH 7.4 PBS buffer. All spectral analysis experiments were performed according to the following procedure. To study the response of the prepared compounds toward various analytes, stock solutions of compounds 1a–1d (2 mM) were prepared in pure DMSO. Stock solutions of cations were obtained by dissolving the chloride salts of the cations (Na^+^, K^+^, Ag^+^, Mg^2+^, Cu^2+^, Ca^2+^, Zn^2+^, Pb^2+^, Cd^2+^, Ba^2+^, Ni^2+^, Hg^2+^, Cl^−^, NO^3−^, HPO_4_^2−^, SO_4_^2−^) in deionized water to afford 20 mM aqueous solutions. Next, 10 µL of the stock solution of the prepared compound was placed in a test tube. Thereafter, 990 µL of the metal-ion solution was added directly. The metal-ion solution was prepared by diluting 10 µL of the 20 mM inorganic salt solution with PBS buffer to a total volume of 990 µL.^25^

#### 2.3.2 Selectivity and sensitivity study of probes 1a–1d

The selectivity of all compounds was investigated using UV-visible spectroscopy, fluorescence spectroscopy, and naked-eye detection in aqueous solutions, following the procedure mentioned above. The measurements were performed on a solution of the probe after the addition of the analyte ions (Na^+^, K^+^, Ag^+^, Mg^2+^, Cu^2+^, Ca^2+^, Zn^2+^, Pb^2+^, Cd^2+^, Ba^2+^, Ni^2+^, Hg^2+^, Cl^−^, NO^3−^, HPO_4_^2−^, SO_4_^2−^). Also, the effect of EDTA, S^2−^, citrate, and lactate on the fluorescence spectra of complexes [1d-Hg^2+^] and [1d-Cu^2+^] was examined. The sensitivity was examined by the absorption and fluorescence titration method using solutions of the probe (10 µM) containing Hg^2+^ and Cu^2+^ in different concentrations.^26^

#### 2.3.3 Detection of metal ions in blood serum samples

Detection of Hg^2+^ ions in blood serum samples was performed by the standard addition method. Initially, the stock solution of probe 1d was prepared at a concentration of 1 mM, while the concentration of the stock solution of Hg^2+^ ions was 100 mM. Then, the blank (1d + blood serum) sample and standard solutions of Hg^2+^ ions (0, 1, 2, 4, 6, 8, 10 µM) spiked with the serum sample were prepared; the standard solutions were mixed with 5 µM of the probe solution diluted with PBS buffer. The absorbance and fluorescence spectra were recorded at 236 nm and 458 nm, respectively. To produce the calibration curve, the absorbance and fluorescence intensities with the Hg^2+^ standard solution (0–10 µM) were measured and plotted, and the linearity (*R*^2^) and recovery% were calculated from the plotted calibration curve.^27,28^

## 3 Results and discussions

### 3.1 UV-visible spectroscopy study of 1a–1d

**Fig. 1:**
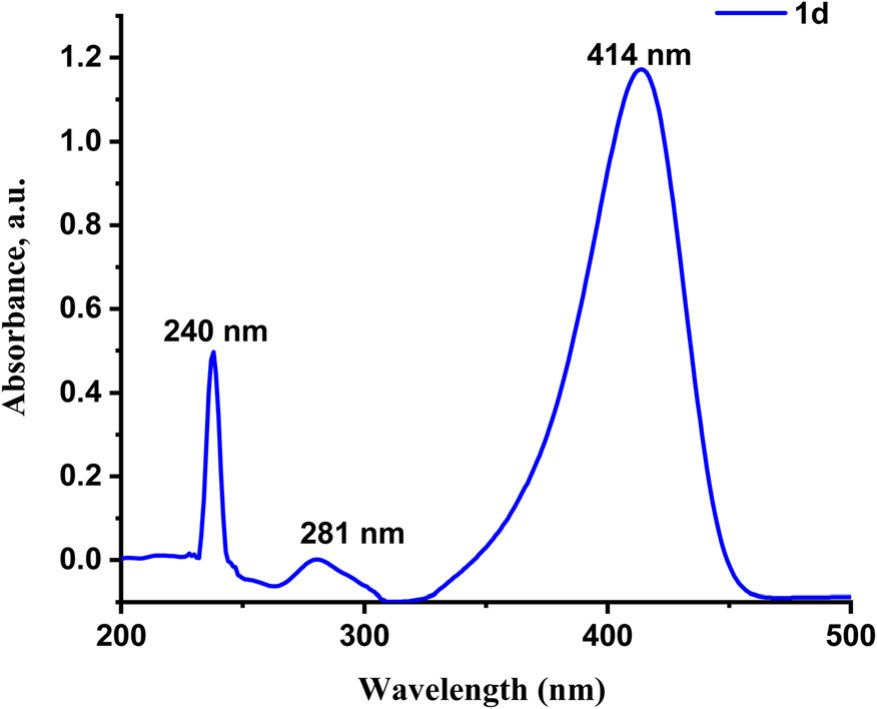
Absorption spectrum of compound 1d (20 µM) in a solution of DMSO : H_2_O (2 : 8 v/v, pH 7.4) at room temperature and the wavelength range of 200–550 nm.

The UV-vis absorption spectra of 1a–1d were recorded to find out how well the prepared sensors respond to UV-vis light. The spectral analysis experiments were carried out in aqueous solutions (deionized water : PBS buffer solution 3 : 1, v/v, pH = 7.4) with probes 1a–1d (20 µM). All prepared probes (1a–1d) exhibited the same behavior (Fig. S1). Probe 1d exhibits high sensitivity and a low detection limit. For probe 1d, three distinct peaks are visible in the UV-vis absorption spectra: the one at the wavelength of 239 nm is due to the conjugated π → π* transition, while the peaks located at 281 nm and 414 nm are due to the n → π* transition, Fig. 1.^29^

**Fig. 2:**
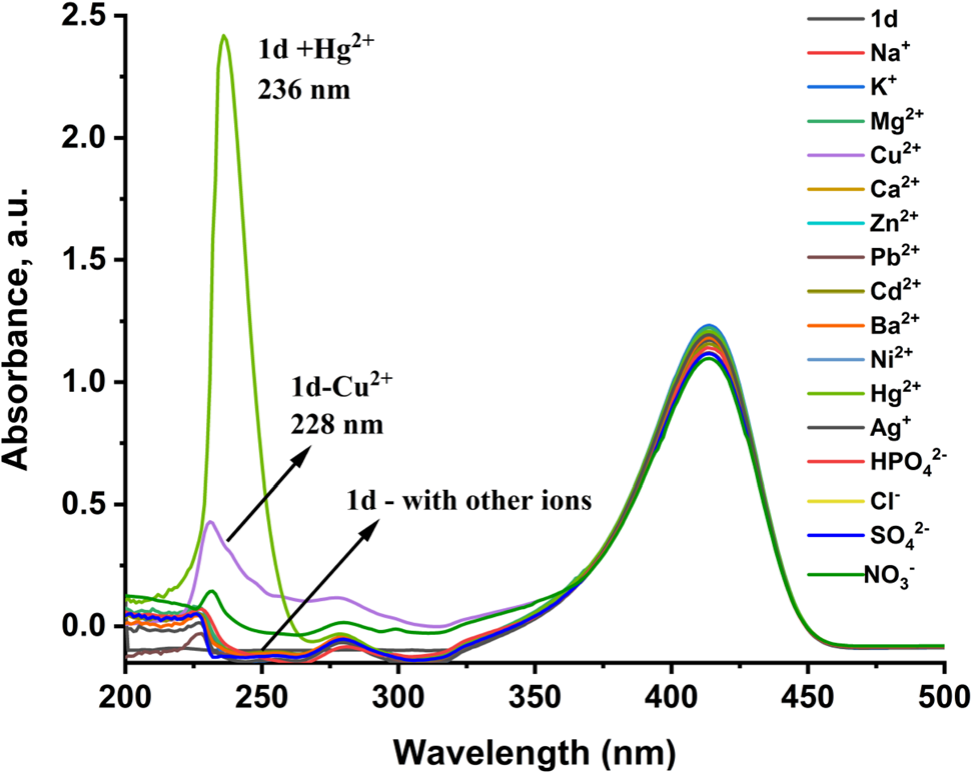
Absorption spectra of the 1d probe response (20 µM) to different metal ions (40 µM) in an H_2_O : PBS buffer solution (3 : 1 v/v and pH = 7.4) at *λ* = 200–500 nm.

On addition of different metal ions (Na^+^, K^+^, Ag^+^, Mg^2+^, Cu^2+^, Ca^2+^, Zn^2+^, Pb^2+^, Cd^2+^, Ba^2+^, Ni^2+^, Hg^2+^, Cl^−^, NO_3_^−^, HPO_4_^2−^, SO_4_^2−^), probe 1d showed a negligible change in the position and intensity of the absorbance peak, except for the Hg^2+^ ion. With the introduction of the Hg^2+^ ion, the peaks at 239 nm and 281 nm disappeared, and a new peak at 236 nm appeared with a higher intensity than the other peaks, as shown in Fig. 2. The peak at 281 nm is attributed to the azomethine group double bond, which breaks and leads to isomerization in the 1d probe. This is the reason for the blue shift from 281 nm to 236 nm.^30,31^ All the prepared probes showed the same selectivity toward the Hg^2+^ ion, but they differed slightly in sensitivity (Fig. S2). Some probes exhibited a more sensitive and selective response to the analyte and higher stability than others. These variations in response may be attributed to differences in their chemical structures and binding affinities for the mercury ion.

The Hg^2+^ ion also changed the color of the 1d probe solution from bright green to yellow upon addition, making it suitable for detection with the naked eye. Also, the color disappeared when the Cu^2+^ ions were added to the 1d probe solution. This color change not only facilitates immediate visual confirmation of the presence of Hg^2+^ and Cu^2+^ ions but also enhances the usability of these probes in field applications where sophisticated detection equipment may not be available (Fig. S3). Consequently, the development of such probes holds significant promise for use in environmental monitoring and safety assessments regarding mercury contamination.

**Fig. 3:**
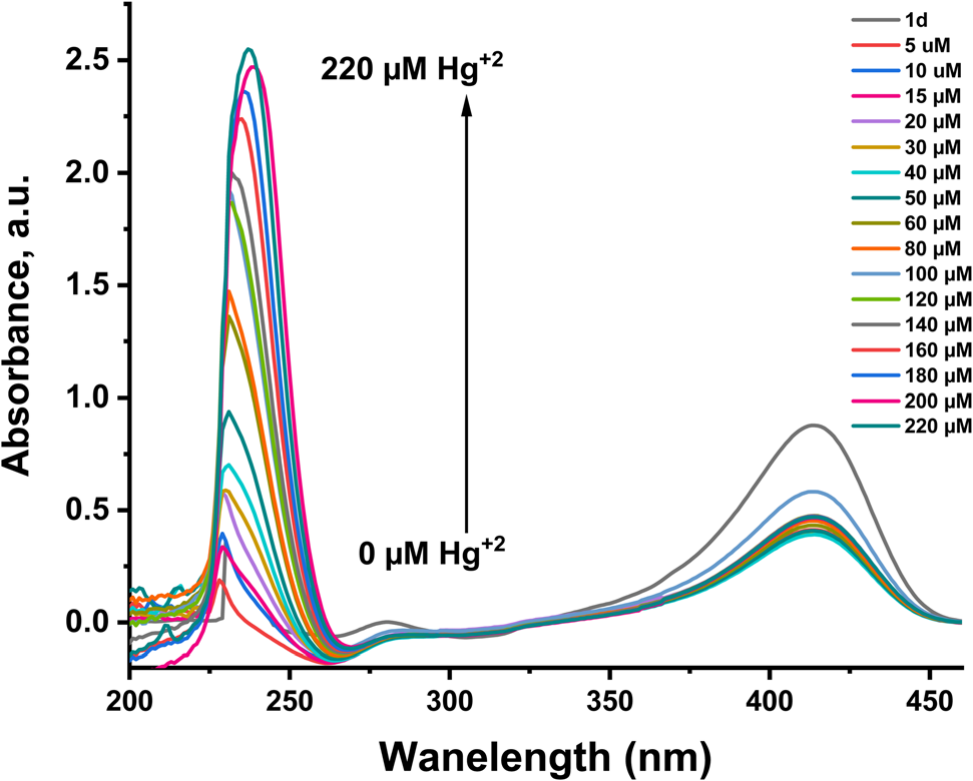
Absorption spectra of probe 1d (20 µM) with gradually increasing the concentration of Hg^2+^ (0–220 µM) in an H_2_O : PBS buffer solution (3 : 1, v/v, pH = 7.4) at *λ* = 236 nm.

To investigate the relationship between the absorption intensity of probe 1d and the Hg^2+^ ion concentration, absorption titration was conducted, which involves the gradual addition of Hg^2+^ ions to probe 1d in the buffer solution, as shown in Fig. 3. The results highlighted a linear relationship from the fitting plot (Fig. S4). This indicates that probe 1d can effectively detect varying concentrations of Hg^2+^ ions, making it a valuable tool for assessing mercury levels in environmental and biological samples. Further studies will explore its application in real-world scenarios to ensure accurate monitoring and timely intervention in contaminated areas. The limit of detection (LOD) is 1.03 × 10^−6^ M. This high sensitivity underscores the potential of probe 1d for environmental monitoring and biological detection, as it can detect even trace amounts of mercury, which is crucial for safeguarding public health. The LOD is acceptable for clinical detection.^32,33^

**Fig. 4:**
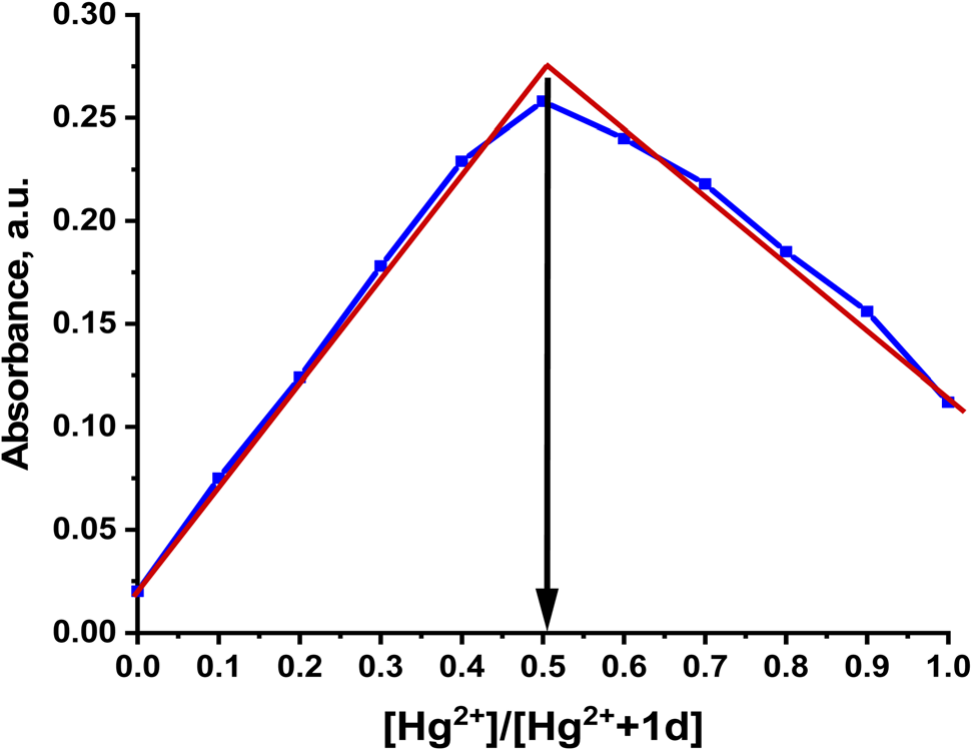
Absorption spectrum of the Job's plot for probe 1d (50 µM) with Hg^2+^ (50 µM) ion at *λ* = 236 nm.

Also, Job's plot analysis was studied to determine the stoichiometry between Hg^2+^ and 1d. The technique involves increasing the molar ratio of Hg^2+^ from 0.1 to 1.0 µM while maintaining the total concentration of 1d and Hg^2+^ at 50.0 µM. As shown in Fig. 4, a 1 : 1 ratio between 1d and Hg^2+^ is confirmed by the absorption maxima at 236 nm, which corresponds to a Hg^2+^ molar fraction of 0.5.

The selectivity of 1d for Hg^2+^ was further investigated by evaluating its selectivity for Hg^2+^ under the same circumstances when other competing metal cations were present; Fig. S5 shows variations in the 1d absorption spectrum. Other competing metal cations were either present or absent when the detection was conducted. These findings clearly show that other competing metal cations do not interfere with Hg^2+^ selective sensing.

### 3.2 Fluorescence spectroscopy study of 1a–1d

**Fig. 5:**
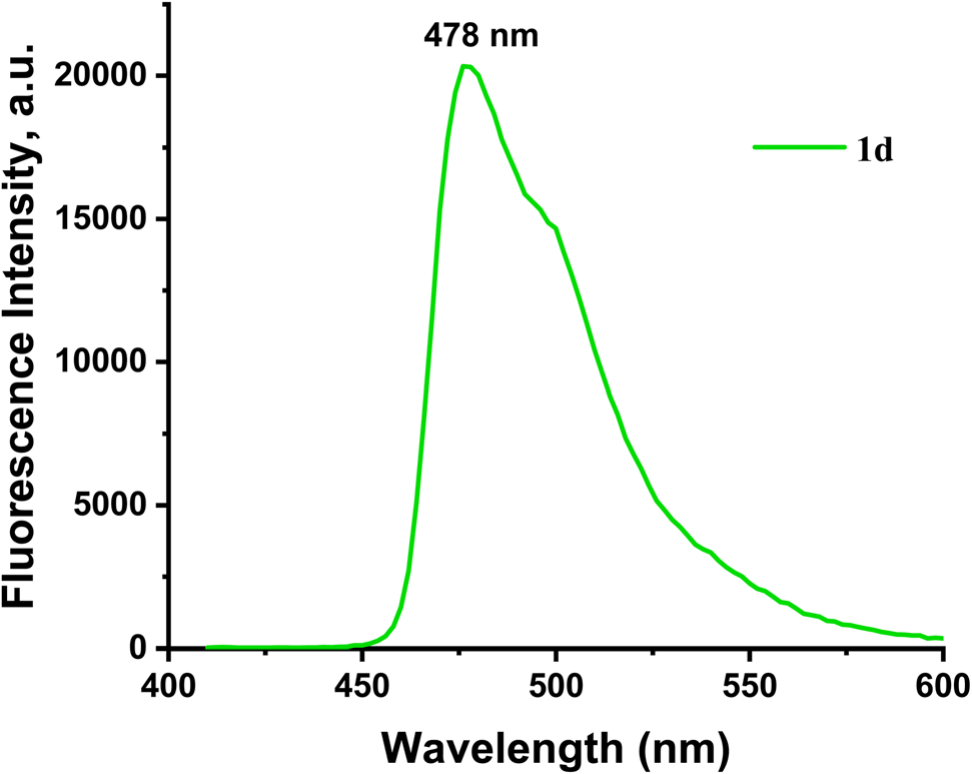
The fluorescence emission spectra of probe 1d (20 µM) in DMSO : H_2_O (2 : 8 v/v and pH = 7.4) at *λ*_ex_ = 402 and *λ*_em_ = 478 nm.

**Fig. 6:**
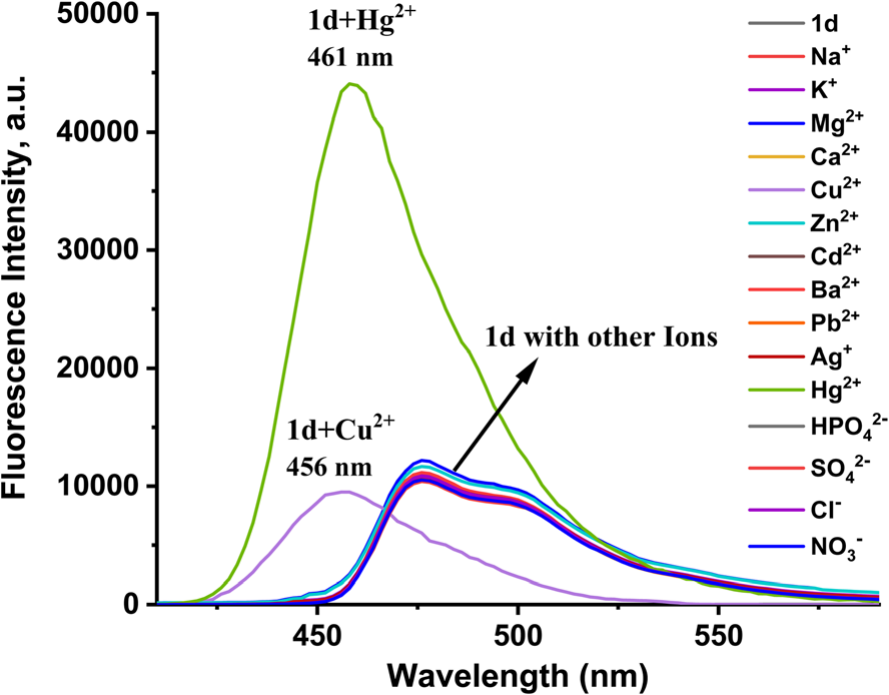
Fluorescence emission spectrum of probe 1d (20 µM) with different ions (40 µM) in an H_2_O : PBS buffer solution (3 : 1 v/v and pH = 7.4) at *λ* = 400–600 nm.

**Fig. 7:**
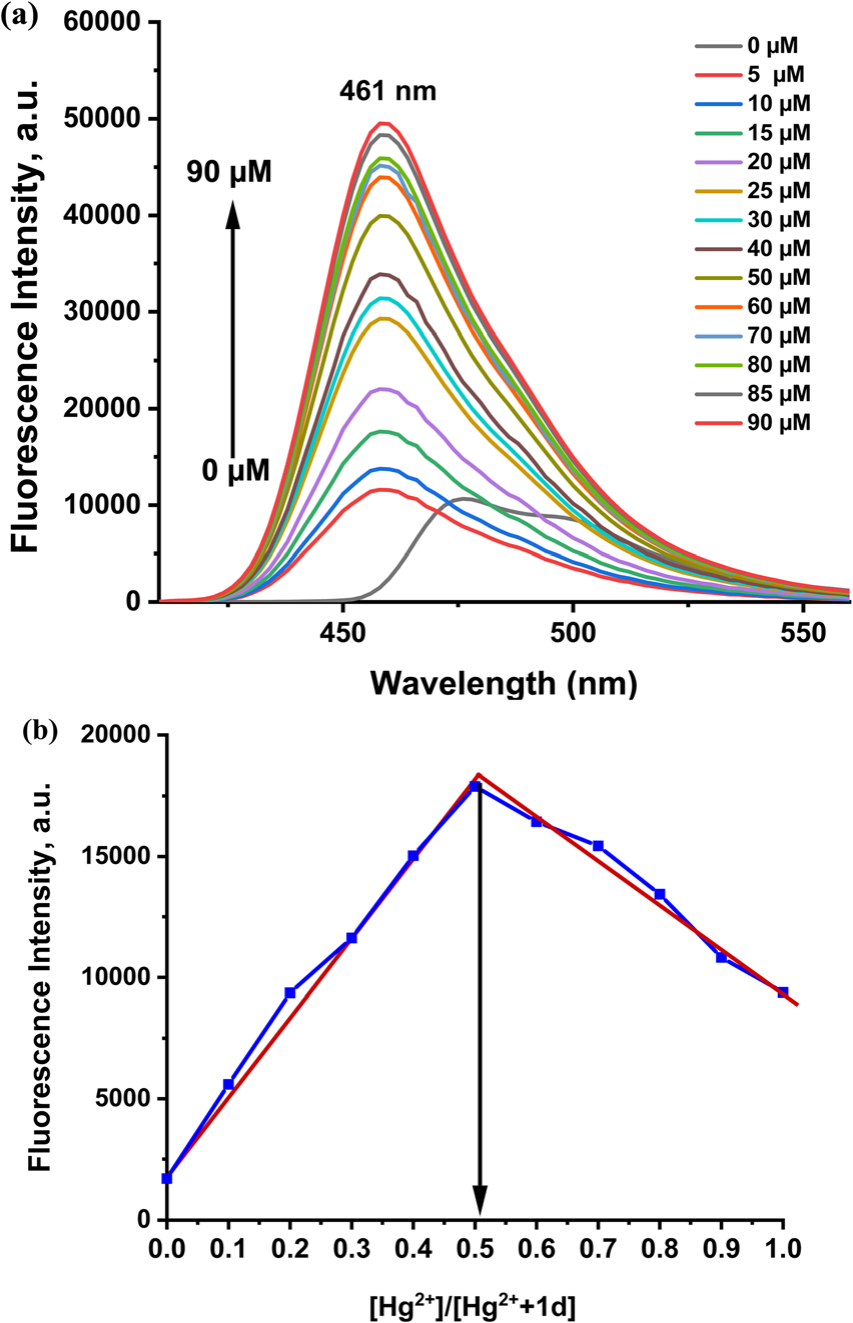
(a) Fluorescence titration of probe 1d (20 µM) with the gradual addition of Hg^2+^ (0–90 µM) at 461 nm. (b) Job's plot for probe 1d (30 µM) with Hg^2+^ (30 µM) at room temperature.

The fluorescence spectra of probes 1a–1d were measured under the same conditions employed for the absorbance measurement, as shown in Fig. S6. After radiation at a wavelength of 402 nm, the probe's fluorescence spectra showed discernible emission. The fluorescence emission maxima peak of 1a–1d appeared at 478–483 nm. As in the absorption spectroscopy case, all prepared probes exhibit the same fluorescence emission behavior, but probe 1d was more sensitive, selective, and stable than the other probes prepared (Fig. 5). Nevertheless, the addition of Hg^2+^ and Cu^2+^ caused a notable change in the emission intensity with a blue shift in the range of 478 nm to 461 nm and 456 nm, respectively. The response of the 1d probe to Hg^2+^ is shown in Fig. 6. The addition of Hg^2+^ ions to the 1d solution leads to fluorescence intensity enhancements. The fluorescence turns on the response toward Hg^2+^ ions through the chelation-enhanced fluorescence (CHEF) pathway. When Hg^2+^ binds to the 1d probe, the electron transfer from the N atom of the imine (CH=N–) group to the conjugated pyrene unit (PET process) is hindered upon coordination with the Hg^2+^ metal ion, as the lone pair on the N atom is coordinated. This results in the chelation-enhanced fluorescence (CHEF).^33^ The binding behavior of the probe with metal ions was investigated by plot fitting of fluorescence titration (Fig.e S7(a)) and Job's analysis Fig. 7(b). As a result, the fluorescence intensity increases with increased concentration of Hg^2+^ ion, with LOD = 36.75 nM and LOQ = 122.5 nM, while the stoichiometry is 1 : 1. Also, the quantum yields of free 1d and [1d-Hg^2+^] were calculated by the comparative Williams method using quinine sulfate (in 0.1 M H_2_SO_4_, *Φ* = 0.54) as the standard, affording 0.039 and 0.43, respectively.

To study the affinity of the probe to Hg^2+^ ions, the Benesi–Hildebrand equation for the 1 : 1 binding ratio was used to determine the association constant (*K*_a_ = 3.5 × 10^7^ M^−1^) value of the 1d-Hg^2+^ complex (Fig. S7(b)).

**Fig. 8:**
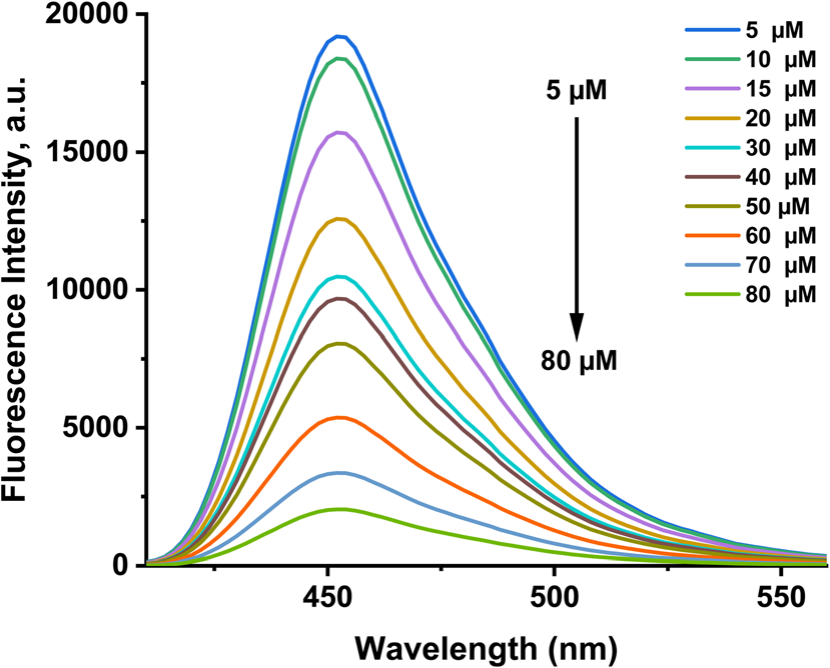
Fluorescence titration of probe 1d (20 µM) with the gradual addition of Cu^2+^ (0–80 µM) at 456 nm.

**Fig. 9:**
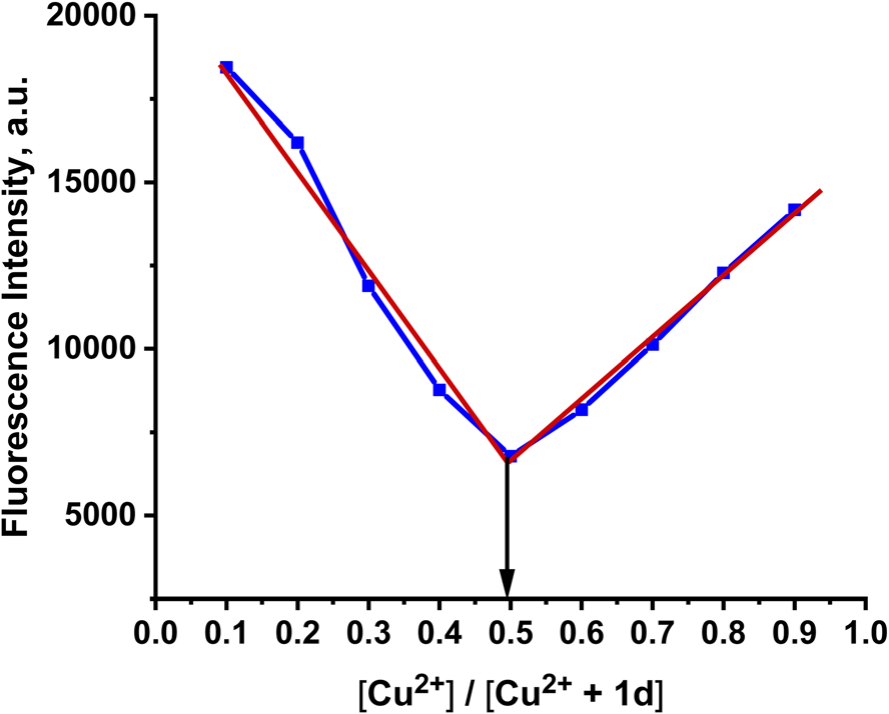
Job's plot for probe 1d (20 µM) with Cu^2+^ (20 µM) at 456 nm.

On the contrary, the addition of Cu^2+^ ions led to a decrease in the fluorescence intensity of the probe, with a blue shift to 456 nm, which means that the binding of the probe with Cu^2+^ ions causes fluorescence quenching with the disappearance of the probe color due to the hypsochromic shift.^34^ The Cu^2+^ ions' paramagnetic characteristics allow them to effectively quench a fluorophore's fluorescence by a photoinduced metal-to-fluorophore electron transfer (PET-Quenching). Furthermore, Cu^2+^ exhibits a quick metal-to-ligand binding kinetics and a particularly high thermodynamic affinity for ligands containing “N” or “O” as chelating elements, among the pertinent paramagnetic metal ions.^35–37^ The fluorescence titration study shows a linear relationship between the fluorescence intensity and the copper-ion concentration, Fig. 8. The fluorescence emission maxima were significantly quenched upon increasing the Cu^2+^ concentration. The interaction of the 1d probe with metal ions was investigated by plot fitting of the fluorescence titration measurement (Fig. S8) and Job's analysis. In Fig. 9, the 1 : 1 binding ratio of the [1d-Cu^2+^] complex formation is shown. The probe exhibits a LOD of about 33 nM and a LOQ of 110 nM. The *K*_a_ value of the 1d-Cu^2+^ complex is 6.9 × 10^6^ M^−1^ (Fig. S8(b)). The fluorescence quantum yield of 1d with Cu^2+^ was found to be 0.001.

**Scheme 2:**
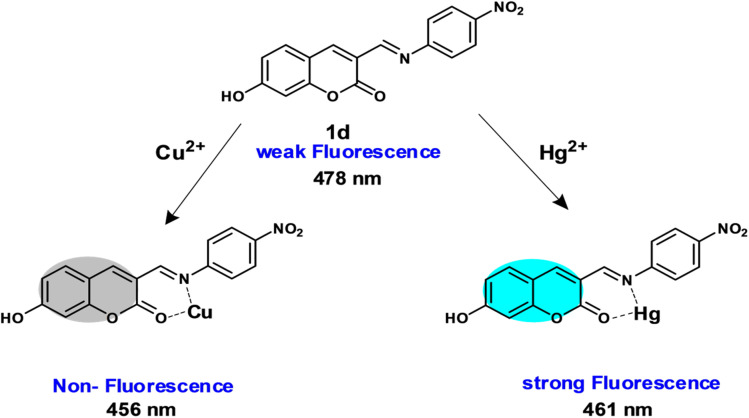
Probable sensing mode of 1d with Hg^2+^ and Cu^2+^ ions.

From FT-IR studies, it was seen that the characteristic stretching vibrational frequencies of coumarin (C=O) shifted slightly to the lower wavenumber from 1719 cm^−1^ to 1709 cm^−1^ (for Hg^2+^) and 1711 cm^−1^ (for Cu^2+^). This demonstrates that the carbonyl group has delocalized the lone pair of electrons on the ester oxygen. Conversely, the stretching vibrational frequencies of imine (C=N) shift slightly to higher wavenumbers, from 1604 cm^−1^ to 1638 cm^−1^ (for Hg^2+^) and 1736 cm^−1^ (for Cu^2+^). This indicates the coordination of the divalent ions and the participation of the single pair of electrons. These studies indicate that both Hg^2+^/Cu^2+^ ions bind to the 1d probe through the oxygen lone pair of carbonyl in the coumarin core and the nitrogen lone pair in the imine group (Fig. S24).^38–40^ The binding site and the sensing mode of probe 1d with Hg^2+^ and Cu^2+^ ions are shown in Scheme 2.

### 3.3 Interference and reversibility of 1a–1d

**Fig. 10:**
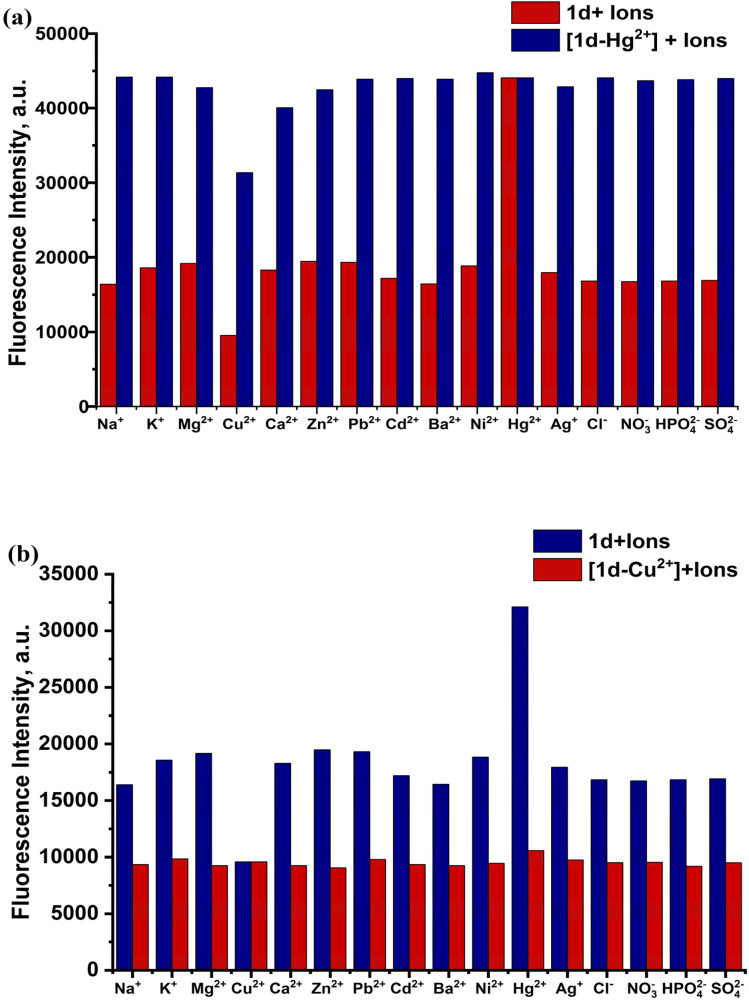
Selectivity of 1d for (a) Hg^2+^in the presence of other metal cations at 461 nm and (b) Cu^2+^ in the presence of other metal cations at 456 nm.

**Fig. 11:**
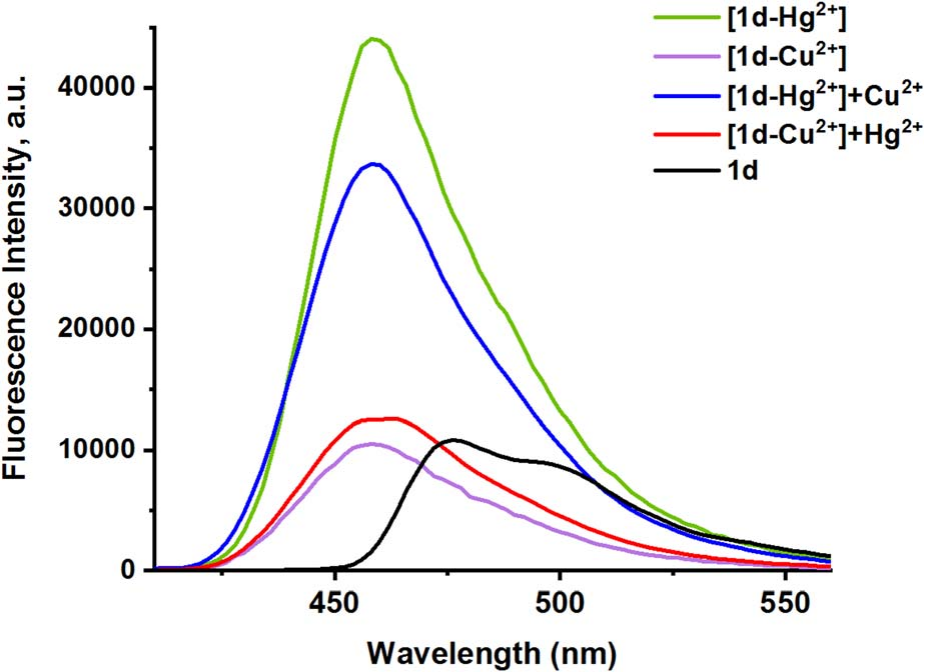
Fluorescence spectra of 1d (1 eq), [1d-Hg^2+^] (2 eq.) in the presence and absence of Cu^2+^ ions (2 eq.), and [1d-Cu^2+^] (2 eq.) in the presence and absence of Hg^2+^ ions (2 eq.).

The selectivity of 1d for Hg^2+^ and Cu^2+^ was further investigated by evaluating its selectivity for Hg^2+^/Cu^2+^ ions under the same conditions, in the presence of other competing cations and anions. Fig. S5 shows variations in the 1d and [1d-Hg^2+^] absorption intensity upon addition of various individual ions (Na^+^, K^+^, Ag^+^, Mg^2+^, Cu^2+^, Ca^2+^, Zn^2+^, Pb^2+^, Cd^2+^, Ba^2+^, Ni^2+^, Hg^2+^, Cl^−^, NO^3−^, HPO_4_^2−^, SO_4_^2−^). These findings clearly show that other competing ions do not interfere with the Hg^2+^ selective sensing. The presence of other ions with the probe causes a non-significant change in the fluorescence intensity and in the peak position. To investigate the effect of interfering ions, the fluorescence emission was studied for a solution of 1d-Hg^2+^ and 1d-Cu^2+^, with the addition of different competing ions. The result shows a negligible effect on the intensity and position of the 1d-Hg^2+^ and 1d-Cu^2+^peaks, as shown in Fig. 10. Meanwhile, the presence of Hg^2+^ and Cu^2+^ together causes an effect in the fluorescence intensity of both individual ion peaks (see Fig. 11).^41,42^ The result indicates the ability of 1d to detect Hg^2+^ and Cu^2+^ at the same time, but the efficiency of quantitative measurement may be affected by the presence of Hg^2+^/Cu^2+^ ions together [43,39].

**Fig. 12:**
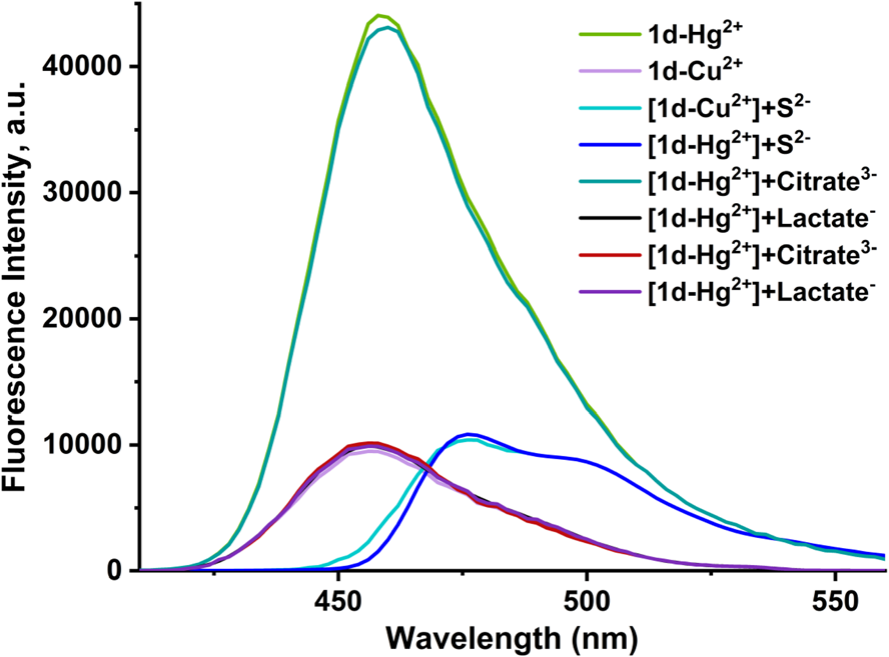
Fluorescence emission spectra of [1d-Hg^2+^] and [1d-Cu^2+^] (10 µM) in the presence of S^2−^, citrate (C_6_H_5_O_7_^3−^) and lactate (C_3_H_5_O_3_^1−^) ions (40 µM).

To improve the selectivity study of probe 1d, the effects of S^2−^, citrate^3−^ and lactate^−^ ions on the selectivity of the 1d were studied. Due to the high affinity of both Hg^2+^ and Cu^2+^ ions for sulfide (S^2−^), which far exceeds their affinity for the binding site on probe 1d, S^2−^ ions are added to the solution of [1d-Hg^2+^] and [1d-Cu^2+^]. This addition reverts the fluorescence emissions to the emission profile of the free probe 1d, as shown in Fig. 11. As a result, S^2−^ can strip the Hg^2+^ and Cu^2+^ metal ions from the [1d-Hg^2+^] and [1d-Cu^2+^] complexes. The metal ions are sequestered into the insoluble sulfide precipitates, HgS and CuS, with astronomically high formation constants. This indicates that the reaction is essentially irreversible, quantitative, and applicable for S^2−^ detection. Conversely, the non-significant effects of citrate and lactate ions on the fluorescence emission of 1d are shown in Fig. 12.^43–45^

**Fig. 13:**
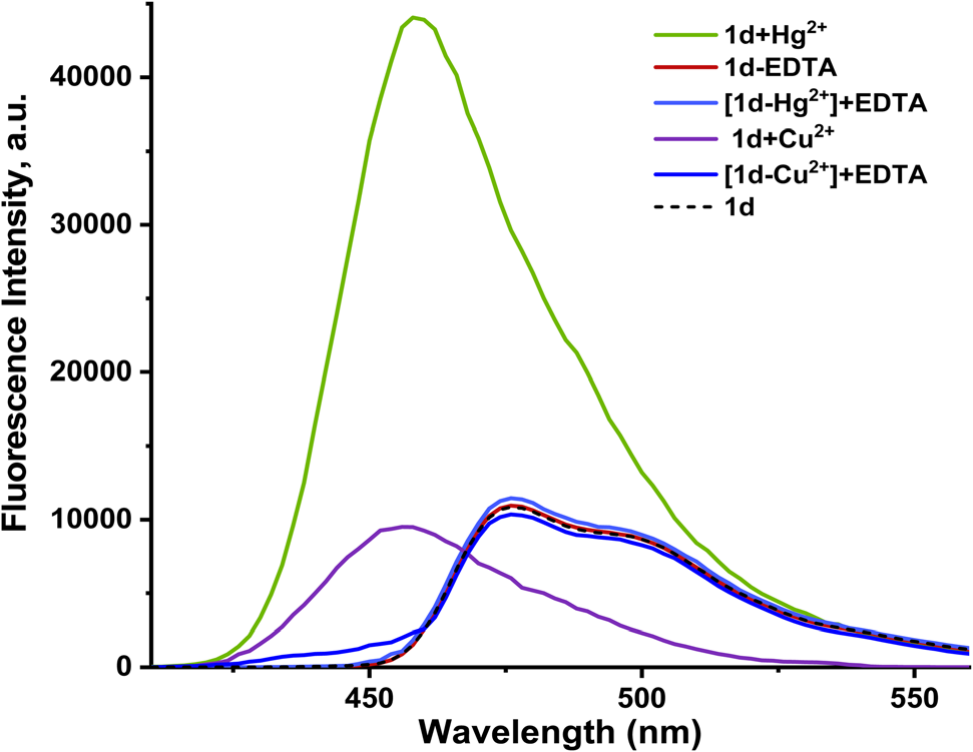
Fluorescence emission spectra of the [1d-Hg^2+^] and [1d-Cu^2+^] ensemble (20.0 µM) in the presence of EDTA (30.0 µM).

**Fig. 14:**
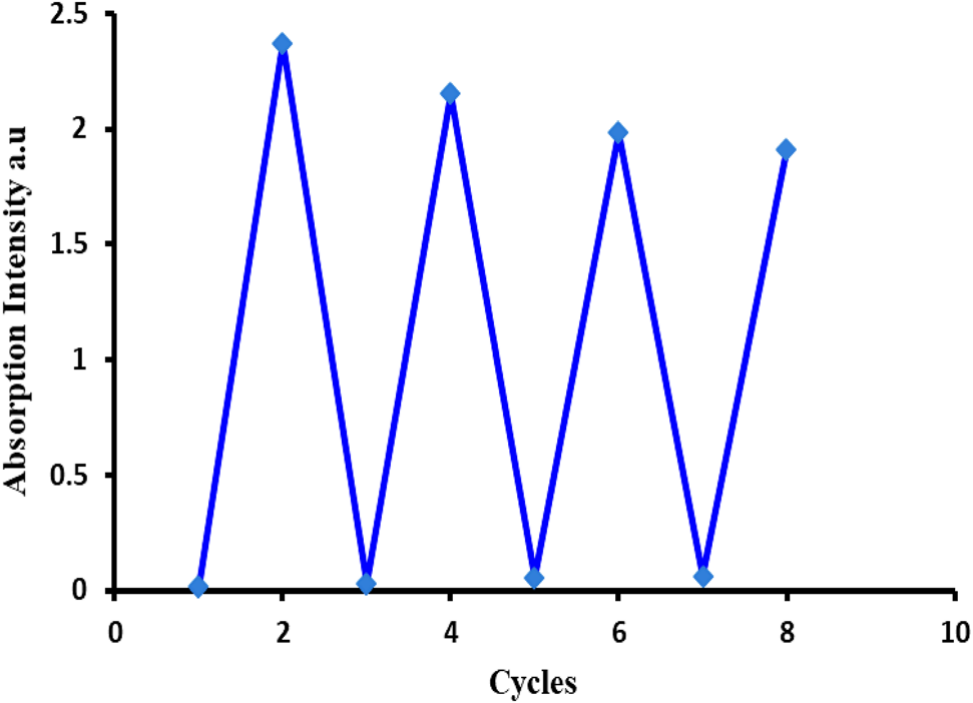
Reversible changes in the absorption intensity of probe 1d at 236 nm after subsequent additions of Hg^2+^ ions and EDTA for 8 cycles.

We also used EDTA as a coordinating ligand to investigate the reversibility characteristics of 1d. Since EDTA is widely accessible and reasonably priced, we chose it as the preferred ligand in this case. With the addition of EDTA (30 µM), the fluorescence intensities of the [1d-Hg^2+^] and [1d-Cu^2+^] complexes were observed to decrease and return to a lower level for 1d, with a red shift to the origin peak, indicating the regeneration of free 1d (Fig. 13). Reports of chemosensors with the same reversibility nature exist.^46,47^ This might be attributed to EDTA's inability to form a preferred compound with Hg^2+^/Cu^2+^ ions and its lack of affinity with 1d. Therefore, free 1d was produced from the complex probe (1d-Hg^2+^) using EDTA. Also, the original absorption peak of [1d-Hg^2+^] was restored upon the addition of EDTA, whereas the addition of Hg^2+^ ions recovered the peak intensity to its maximum. In addition, 1d exhibited a good fluorescence response for at least 8 cycles when Cu^2+^/Hg^2+^ and EDTA were introduced alternately (see Fig. 14). This indicates that the probe can be utilized again for more Hg^2+^/Cu^2+^ sensing with good cycle reversibility in real samples.^38^

### 3.4 Response time and pH effect of 1a–1d in the presence of Hg^2+^ and Cu^2+^

**Fig. 15:**
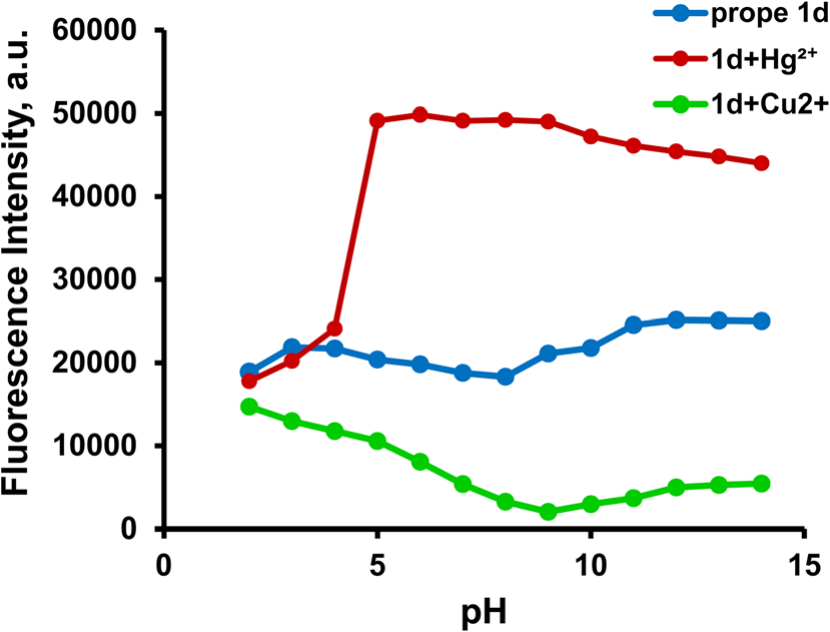
Effect of pH on the fluorescence intensity of probe 1d (20 µM) in DMSO : H2O (2 : 8 v/v and pH = 7.4) alone at 478 nm and in the presence of Hg^2+^ at 461 nm and Cu^2+^ at 456 nm.

**Scheme 3:**
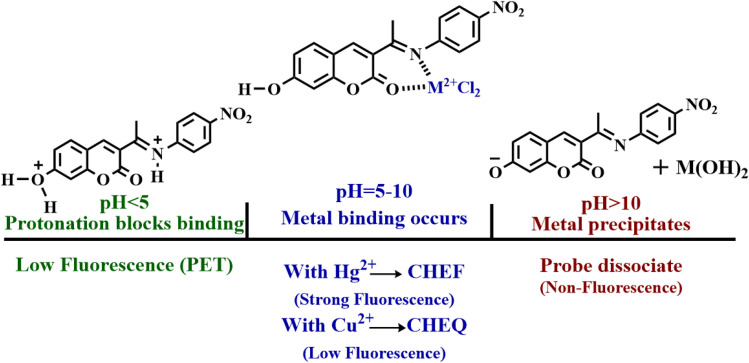
The proposed mechanism for the pH-dependent fluorescence response of the probe in acidic, neutral, and basic media.

The fluorescence response profiles offer important information on the bounds of applications and operational sensing systems. The pH sensitivity was examined both with and without Hg^2+^ and Cu^2+^, across the pH range of 2–12 (see Fig. 15). The protonation state of the donor atoms (phenolic –OH, imine –N=) and the stability of the metal ions in solution govern the fluorescence of 1d, [1d-Hg^2+^], and [1d-Cu^2+^]. Probe 1d shows a low fluorescence intensity, due to an effective photoinduced electron transfer (PET) mechanism from the imine nitrogen lone pair to the heated coumarin core. In strong acidic media (pH = 2–4), the key donor atoms of the probe are protonated, the phenolic oxygen becomes –OH_2_^+^ and the imine nitrogen becomes =NH^+^. This protonation has critical effects: It blocks the receptor site, making it incapable of coordinating to metal ions. The result is that the fluorescence remains in a deeply quenched state even in the presence of Hg^2+^ and Cu^2+^ ions.^19,21,48,49^ The fluorescence intensity sharply increases in the presence of the Hg^2+^ ion in the pH range of 5.0–10.0 in comparison to the free ligand. Above pH 5, effective chelation takes place through the imine and carbonyl group donors, resulting in a noticeable “turn-on” reaction because of PET suppression and structural rigidification. The emission gradually decreases at high pH values (>10), due to interference from hydroxide-bound Hg^2+^ species, such as Hg(OH)_2_ and Hg(OH)_4_^2−^, which compete with probe binding. Other pH-dependent Hg^2+^-sensing systems exhibit the same behavior.^21,50^ Similarly, the 1d-Cu^2+^ complex shows a substantial quenching effect between pH 5 and pH 10.^51^ The primary causes of this quenching are ligand-to-metal charge transfer (LMCT) and paramagnetic relaxation of Cu^2+^, which effectively deactivates the excited state of the coumarin fluorophore.^20^ Cu^2+^ ions have a tendency to precipitate as Cu(OH)_2_ or form polynuclear hydroxide complexes at high pH values (>10), which lowers their effective concentration in solution. As a result, quenching is somewhat reduced, which causes the fluorescence intensity to slightly recover.^19^ These results indicate that the 1d pH-dependent dual response can be used as a selective fluorescent probe with good sensing ability to recognize and distinguish Hg^2+^ and Cu^2+^. This performance is achieved over a wide pH range of 5.0–10.0 (encompassing the physiological pH conditions for bioanalysis applications) and in the presence of other competing metal ions. Scheme 3 shows the proposed mechanism for probe 1d’s pH-dependent fluorescence response. PET quenching is maintained at acidic pH levels *via* protonating donor sites, which inhibits metal binding. At neutral pH values, binding with Hg^2+^ prevents PET and causes strong fluorescence (CHEF), but Cu^2+^ causes fluorescence quenching (CHEQ). The complexes are dissociated by metal hydroxide precipitation under alkaline pH, which lowers the fluorescence intensity.

**Fig. 16:**
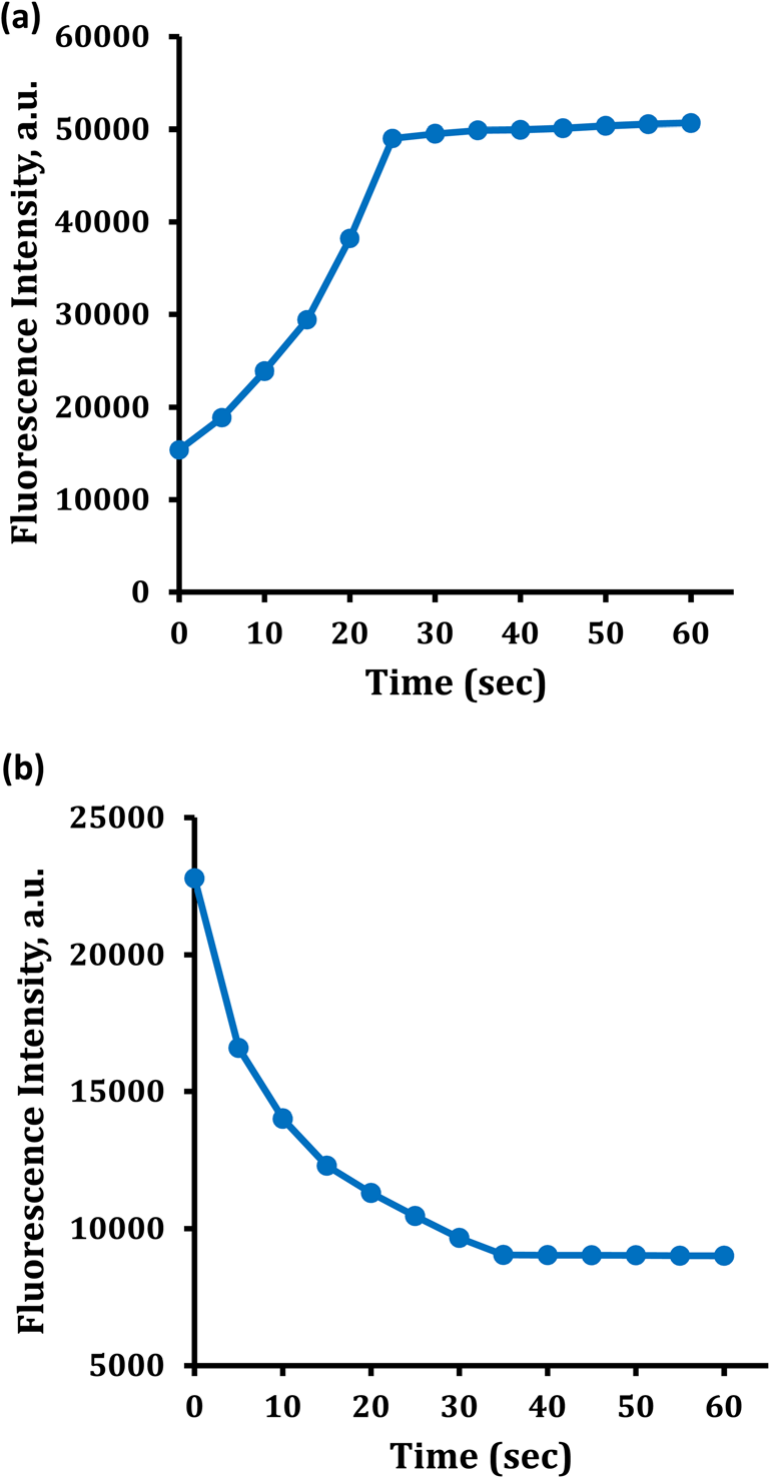
Fluorescence response of 1d in the presence of (a) Hg^2+^ ion and (b) Cu^2+^ at different times (5–60 s).

In the presence of Hg^2+^ and Cu^2+^ ions, the time-dependent absorbance and fluorescence intensity profiles of 1d were measured. Three distinct Hg^2+^ and Cu^2+^ ion concentrations were observed in these studies. When Hg^2+^ ions are present, the absorbance and fluorescence intensities exhibit an almost instantaneous increase. Due to a significant complexation of 1d with Hg^2+^ ions *via* O and N atoms, which may be explained by photoinduced electron transfer, the absorbance and fluorescence intensities increase with time to a particular point at about 30–35 seconds, Fig. 16(a). Then, they stay nearly constant for more than 1 hour. On the other hand, the fluorescence intensity quenching behavior was observed quickly in the presence of Cu^2+^ ions, Fig. 16(b). Once more, the intensity of the fluorescence drops with time until it reaches a fixed point after 40 seconds and stays there for over 1 hour. Strong metal-fluorophore communication, which causes fluorescence quenching, might be the cause of this drop in the fluorescence intensity. The results of these experiments demonstrate the rapid reaction times of the probe for Hg^2+^ and Cu^2+^ ion monitoring, separately, which can be applied to various real-world applications.

### 3.5 Comparative studies with reported probes

Although many studies have explored the development of fluorescent probes for the dual detection of Hg^2+^ and Cu^2+^, many of these probe systems have limitations, such as slow response times, irreversibility, insufficient sensitivity for real-world applications, and a lack of validation for complex real-world samples. In order to overcome these obstacles, the coumarin-based chemosensor 1d described in this study incorporates several key innovations that distinguish it from its competitors. The incorporation of a potent electron-withdrawing nitro group modifies the probe's electrical characteristics, resulting in enhanced sensitivity with detection limits in the nanomolar range (36.75 nM for Hg^2+^, 33 nM for Cu^2+^), matching or outperforming the majority of modern dual probes (Table 1). Its usefulness for field analysis, where equipment may not be accessible, is increased by the additional property of Hg^2+^/Cu^2+^ being detectable with the naked eye (color shift from green to yellow). A critical novelty lies in its practical applicability: 1d is effectively used for the precise detection of Hg^2+^ and Cu^2+^ in human blood serum, with outstanding recovery rates (90–99.9%), whereas many probes are only proven in pure aqueous buffers. As a necessary prerequisite for therapeutic or environmental applications, this functionality highlights the resilience of 1d against complex biological matrices. Additionally, compared to single-use, irreversible chemosensors, the probe's reversibility, as shown over several cycles using EDTA, provides a reusable and affordable sensing platform. Probe 1d’s novelty lies not only in its dual-detection capabilities but also in its speed, remarkable sensitivity, reversible operation, and proven effectiveness in a genuine biological sample. Probe 1d is positioned as an excellent and useful instrument for advanced environmental monitoring and biomedical diagnostics due to its multifarious performance.

**Table 1:** Comparative performance of probe 1d and previously reported fluorescence dual chemosensors for Hg^2+^ and Cu^2+^ detection

Probe (code)	Target ion	Detection limit	*K*_a_	Medium	Response time	Reversibility/pH range	Ref.
2-OxI-Rh	Hg^2+^ (fluorescence)	3.36 × 10^−6^ M	2.15 × 10^4^ M^−1^	Aqueous buffer, organic solvent	ND	ND/pH = 7.0	38
	Cu^2+^ (fluorescence)	2.31 × 10^−6^M	1.21 × 10^4^ M^−1^				
R6GCP	Hg^2+^ (UV-vis)/(fluorescence)	1.24 × 10^−5^ M/1.32 × 10^−8^ M	6.32 × 10^4^ M^−1^	Aqueous	ND	ND/pH = 6–7	52
	Cu^2+^ (UV-vis)/(fluorescence)	5.29 × 10^−6^ M/1.91 × 10^−7^ M	2.92 × 10^7^ M^−1^				
AQ	Hg^2+^ (fluorescence)	2.1 × 10^−9^ M	ND	Aqueous		Yes/pH = ND	53
	Cu^2+^ (UV-vis)	ND	ND				
PEGSRh	Hg^2+^ (fluorescence)	2.85 × 10^−6^ M	1.63 × 10^5^ M^−1^	Aqueous	10 s	Yes/pH = 5–10	54
	Cu^2+^ (UV-vis)	5.92 × 10^−7^ M	5.38 × 10^4^ M^−1^	Real water sample		Yes pH = 4–10	
FAHK	Hg^2+^	20.3 × 10^−9^ M	1.68 × 10^6^M^−2^	Aqueous living cell	50–350 s	Yes/pH = 6–12	44
	Cu^2+^ (fluorescence) (and S^2^ cascade)	29.8 × 10^−9^ M	1.19 × 10^7^M^−2^	Imaging stripe			
1d	Hg^2+^ (UV-vis)/(fluorescence)	36.75 × 10^−9^ M/1.03 × 10^−6^ M	3.5 × 10^7^ M^−1^	Aqueous buffer solution	30	Yes/pH = 5–10	This work
	Cu^2+^ (fluorescence) (S^2−^ cascade)	33 × 10^−9^ M	6.9 × 10^6^ M^−1^	Blood serum sample. Naked eye detection	35 s	Yes/pH = 5–10	

### 3.6 Hg^2+^ detection in blood serum sample

Probe 1d’s performance was evaluated for the measurement of Hg^2+^ and Cu^2+^ in human blood serum in order to determine its practical application. When compared to the probe blank, the examination of an unspiked serum sample revealed a slight change in the absorption and fluorescence signals, suggesting that the endogenous concentration of Hg^2+^ was below the detection limit of the technique. Independent clinical examination found that the endogenous levels of Cu^2+^ in the same sample were 9 µM. Therefore, the accuracy and matrix tolerance of the probe were verified using the conventional addition technique. Hg^2+^ and Cu^2+^ at defined amounts (0–10 µM) were added to the serum samples. The fluorescence intensity showed a linear response to both Hg^2+^ and Cu^2+^ contents, while the absorption intensity showed a linear response to the Hg^2+^ content. The methodology's outstanding accuracy was demonstrated by its high percentage recoveries and strong linearity (high *R*^2^ values), as detailed in Table 2 and Fig. S9–11. These results demonstrate the considerable potential of chemosensor 1d for diagnostic and environmental monitoring applications by confirming that it offers a dependable and accurate method for the simultaneous detection and measurement of Hg^2+^ and Cu^2+^ in complex biological matrices, like blood serum.

**Table 2:** Recovery test of Hg^2+^ and Cu^2+^ ions spiked in human blood serum samples by UV-vis and fluorescence spectroscopy^a^

Spiked concentration (µM)	Found absorption (µM)	Recovery % (absorption)	Found fluorescence (µM)	Recovery % (fluorescence)
Hg^2+^				
0	—	—	—	—
2	1.804 ± 0.60	90.0	1.822 ± 0.42	91.1
4	3.808 ± 0.75	95.0	3.845 ± 0.44	96.1
6	5.85 ± 0.81	97.5	5.91 ± 0.57	98.5
8	7.96 ± 1.01	99.5	7.985 ± 0.61	99.8
10	9.98 ± 1.09	99.8	9.99 ± 0.88	99.9

Cu^2+^				
0			9	—
2			10.903 ± 0.45	99.11
4			12.923 ± 0.48	99.40
6			14.910 ± 0.65	99.40
8			16.923 ± 0.74	99.54
10			18.997 ± 0.91	99.98

a Mean value (*n* = 3) ± standard deviation.

## 4 Conclusions

This work effectively developed and synthesized a series of new coumarin-based chemosensors (1a–1d) for the dual detection of Hg^2+^ and Cu^2+^ ions in biological and aquatic environments. Probe 1d (nitro derivative) performed exceptionally well, showing two unique optical responses: a quenching effect for Cu^2+^ due to paramagnetic interactions at 456 nm and a fluorescence “turn-on” for Hg^2+^*via* chelation-enhanced fluorescence (CHEF) at 461 nm. The probe outperformed numerous current sensors, exhibiting exceptionally low detection limits (36.75 nM for Hg^2+^ and 33 nM for Cu^2+^) and quick reaction times (<35 seconds). Reversibility experiments with EDTA highlighted the probe's reusability, while selectivity experiments verified that competing metal ions caused little interference. Notably, 1d made it possible to detect Hg^2+^ with the naked eye by causing a noticeable color shift from green to yellow. Hg^2+^ and Cu^2+^ measurement exhibited good recovery rates of 90–99.8% in absorption spectra and 91–99.9% in fluorescence spectra for Hg^2+^ and 99.1–99.9% for Cu^2+^, with strong linearity, confirming the practical applicability of the probe in human blood serum. These results demonstrate the promise of coumarin-based probes as economical, effective instruments for clinical diagnostics and real-time environmental monitoring, especially in environments with limited resources.

## Ethical statement

All experimental procedures were performed in accordance with the guidelines of the Ethical Approval Committee of the University of Anbar and were approved by the Ethical Committee at the University of Anbar. Informed Consent were obtained from the human participants of this study.

## Conflicts of interest

There are no conflicts to declare.

## Supplementary Material

RA-015-D5RA05643H-s001

## Data Availability

All relevant data supporting the findings of this study are included within the article and its supplementary information (SI). Supplementary information: additional spectroscopic data (^1^H NMR, ^13^C NMR, FT-IR), UV-Vis and fluorescence spectra for probes 1a–1c, Job's plot analyses, titration curves for binding constant calculations, and further interference study data. See DOI: https://doi.org/10.1039/d5ra05643h.
