# Supplementary material for: Design and synthesis of new coumarin-based fluorescent chemosensors for the dual detection of Hg2+ and Cu2+ in aqueous and biological samples
Source: RSC Adv. 2025 Nov 17;15(52):44611–22. doi: 10.1039/d5ra05643h (PMC12621305; doi:10.1039/d5ra05643h)
Supplement: RA-015-D5RA05643H-s001 [file RA-015-D5RA05643H-s001.pdf]

This journal is © The Royal Society of Chemistry 2025

Supplementary File

Design and Synthesis of New Coumarin-Based Fluorescent Chemosensors for Dual Detection of Hg^{2+} and Cu^{2+} in Aqueous and Biological Samples

Wahaj Raed Abbas[1] and Mohammed Abed Kadhim[2]

[1] Nanomaterial Research Center, University of Anbar https://orcid.org/0009-0009-0525-4425

[2] Department of Chemistry, College of Education for Pure Sciences, University of Anbar, Iraq https://orcid.org/0000-0001-9096-7642

Figure Captions:

**Figure S1**	Absorption spectra of compounds (a) **1a**, (b) **1b**, (c) **1c** and (d)**1d** (20 µM) in solution of DMSO: H_2O (2:8 v/v, pH 7.4) at room temperature and the wavelength range 200-550 nm.
**Figure S2**	Absorption spectra of the **1a-1d** probe response (20 µM) to different metal ions (40 µM) in H_2O: PBS buffer solution (3:1, v/v, pH=7.4) at λ= 200-500 nm.
**Figure S3**	Photographs depicting distinct behavior of **1d** in the presence of Hg^{2+}, Cu^{2+}, and other ions.
**Figure S4**	Fitting plot of absorption titration for Hg^{2+} ion (0-220 µM) with **1d** probe (20 µM) at λ= 236 nm.
**Figure S5**	Selectivity of **1d** probe for Hg^{2+} in the presence of other metal cations.
**Figure S6**	The fluorescence emission spectra of compounds (a) **1a** (b) **1b**, (c)**1c**, and (d) **1d** (20 µM) in solution of DMSO:H_2O (2:8 v/v, pH=7.4) at room temperature and wavelength range 400-600 nm.
**Figure S7**	(a) Fitting plot of linear relationship of probe 1d with Hg^{2+} concentration; (b) Plot of ($1/(F-F_o)$) vs ($1/Hg^{2+}$).
**Figure S8**	(a) Fitting plot of linear relationship of probe 1d with Cu^{2+} concentration; (b) Plot of ($1/(F-F_o)$) vs ($1/Cu^{2+}$).
**Figure S9**	(a) Absorption calibration curve of **1d** (10 µM) with Hg^{2+} ion (0-10 µM), (b) Absorption intensity of **1d** (10 µM) with spiked Hg^{2+} ion (0, 2, 4, 6, 8, 10 µM) in serum blood sample.
**Figure S10**	(a) Fluorescence calibration curve of **1d** (10 µM) with Hg^{2+} ion (0-10 µM), (b) Fluorescence intensity of **1d** (10 µM) with spiked Hg^{2+} ion (0, 2, 4, 6, 8, 10 µM) in serum blood sample .
**Figure S11**	(a) Fluorescence calibration curve of **1d** (10 µM) with Cu^{2+} ion (0-20µM), (b) Fluorescence intensity of **1d** (10 µM) with spiked Cu^{2+} ion (0, 2, 4, 6, 8, 10 µM) in serum blood sample .
**Figure S12**	^{1}H-NMR-spectrum of compound 1a.

**Figure S13**	^{13}C-NMR-spectrum of compound 1a.
**Figure S14**	FT-IR spectrum of compound 1a.
**Figure S15**	^{1}H-NMR-spectrum of compound 1b.
**Figure S16**	^{13}C-NMR-spectrum of compound 1b.
**Figure S17**	FT-IR spectrum of compound 1b.
**Figure S18**	^{1}H-NMR-spectrum of compound 1c.
**Figure S19**	^{13}C-NMR-spectrum of compound 1c.
**Figure S20**	FT-IR spectrum of compound 1c.
**Figure S21**	^{1}H-NMR-spectrum of compound 1c.
**Figure S22**	^{13}C-NMR-spectrum of compound 1c.
**Figure S23**	FT-IR spectrum of compound 1c.
**Figure S24**	FT-IR spectrum of (a) probe 1d-Hg^{2+}, (b) 1d-Cu^{2+}.

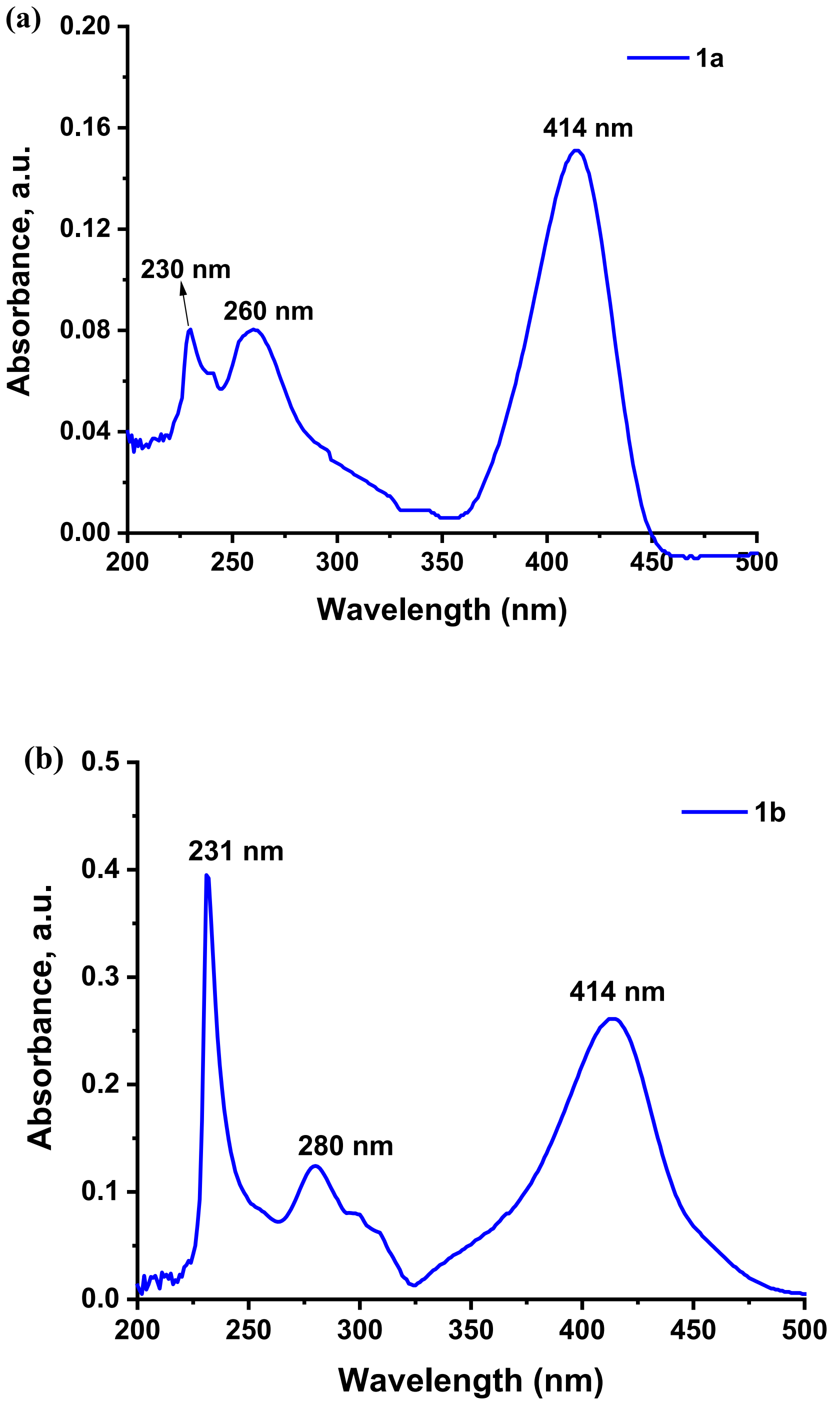
(a)
1a
414 nm
230 nm
260 nm
Absorbance, a.u.
0.20
0.16
0.12
0.08
0.04
0.00
200
250
300
350
400
450
500
Wavelength (nm)
(b)
1b
231 nm
280 nm
414 nm
Absorbance, a.u.
0.5
0.4
0.3
0.2
0.1
0.0
200
250
300
350
400
450
500
Wavelength (nm)

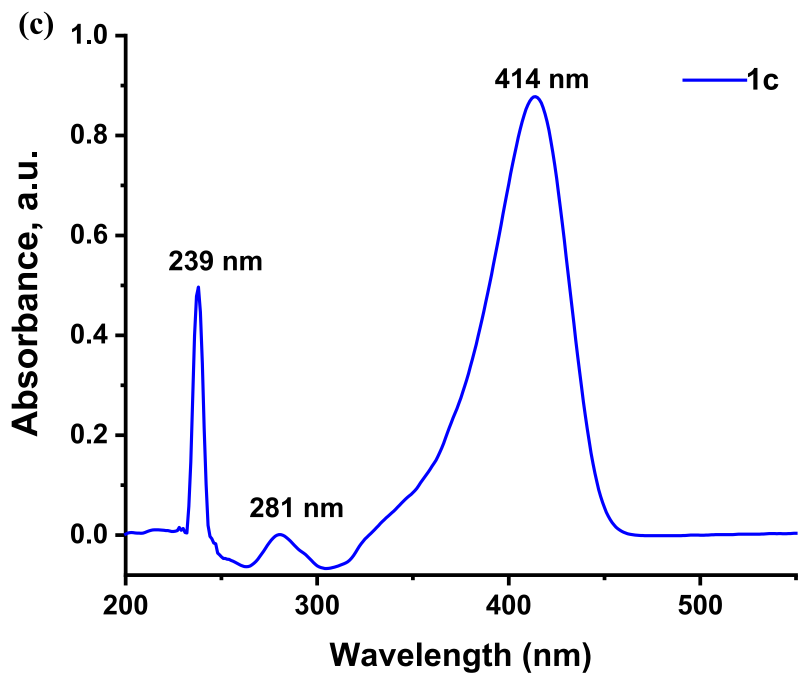

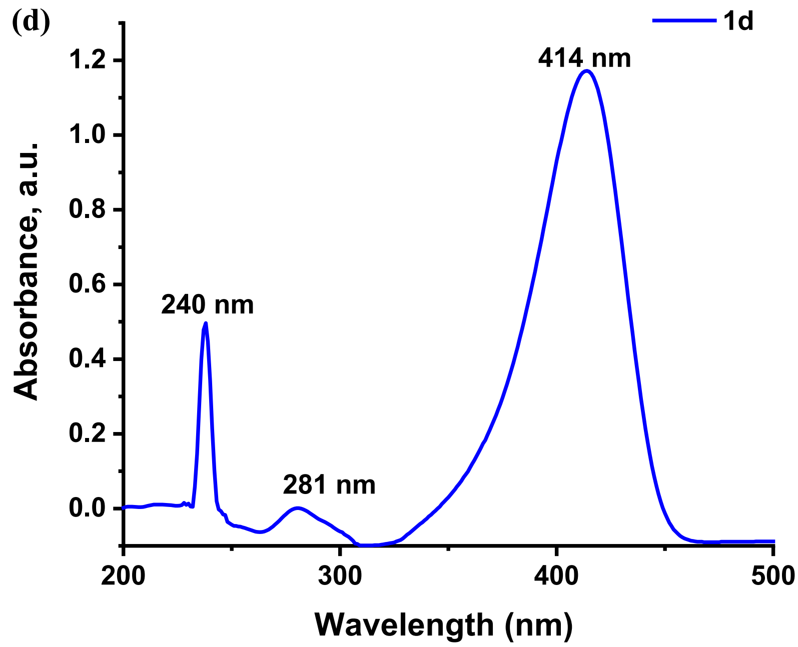

Figure S1: Absorption spectra of compounds (a) **1a**, (b) **1b**, (c) **1c** and (d)**1d** (20 μM) in solution of DMSO: H_2O (2:8 v/v, pH 7.4) at room temperature and the wavelength range 200-550 nm.

(a)

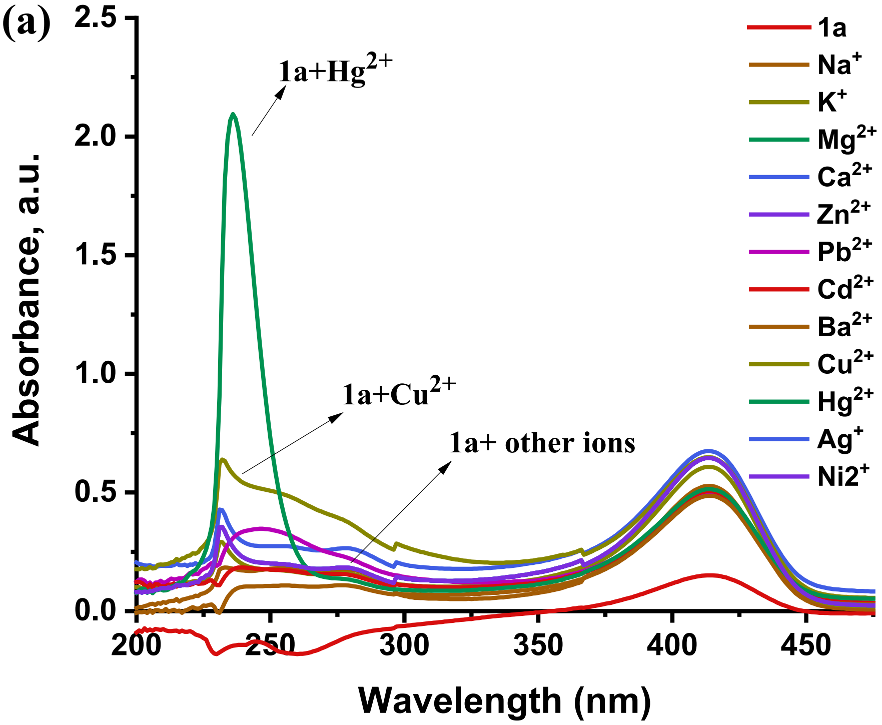
1a+Hg2+
1a+Cu2+
1a+ other ions
Absorbance, a.u.
Wavelength (nm)
1a
Na+
K+
Mg2+
Ca2+
Zn2+
Pb2+
Cd2+
Ba2+
Cu2+
Hg2+
Ag+
Ni2+

(b)

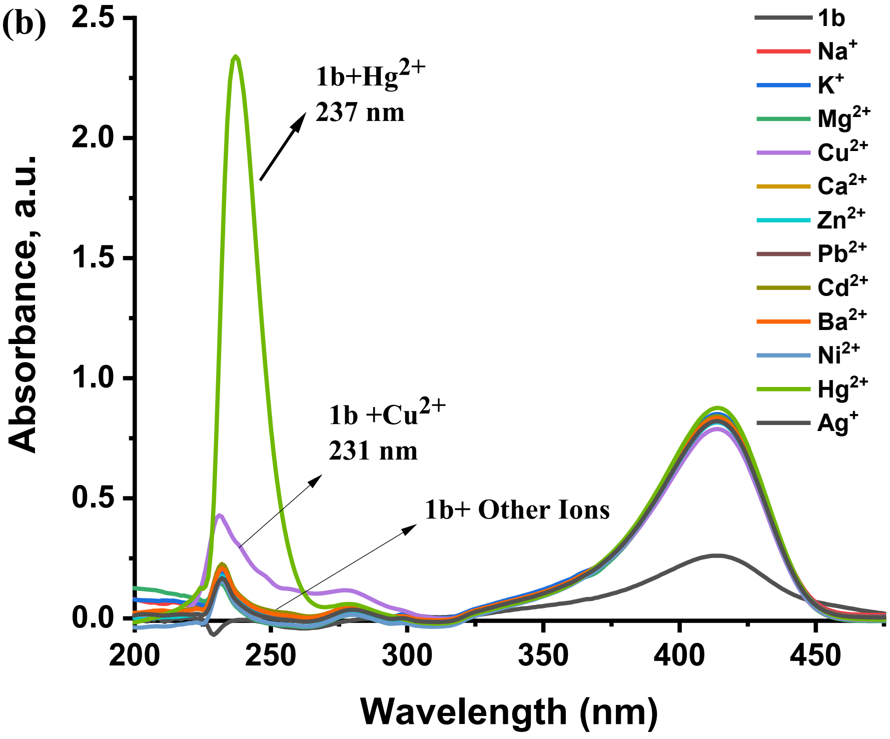
1b+Hg2+
237 nm
1b +Cu2+
231 nm
1b+ Other Ions
Absorbance, a.u.
Wavelength (nm)
1b
Na+
K+
Mg2+
Cu2+
Ca2+
Zn2+
Pb2+
Cd2+
Ba2+
Ni2+
Hg2+
Ag+

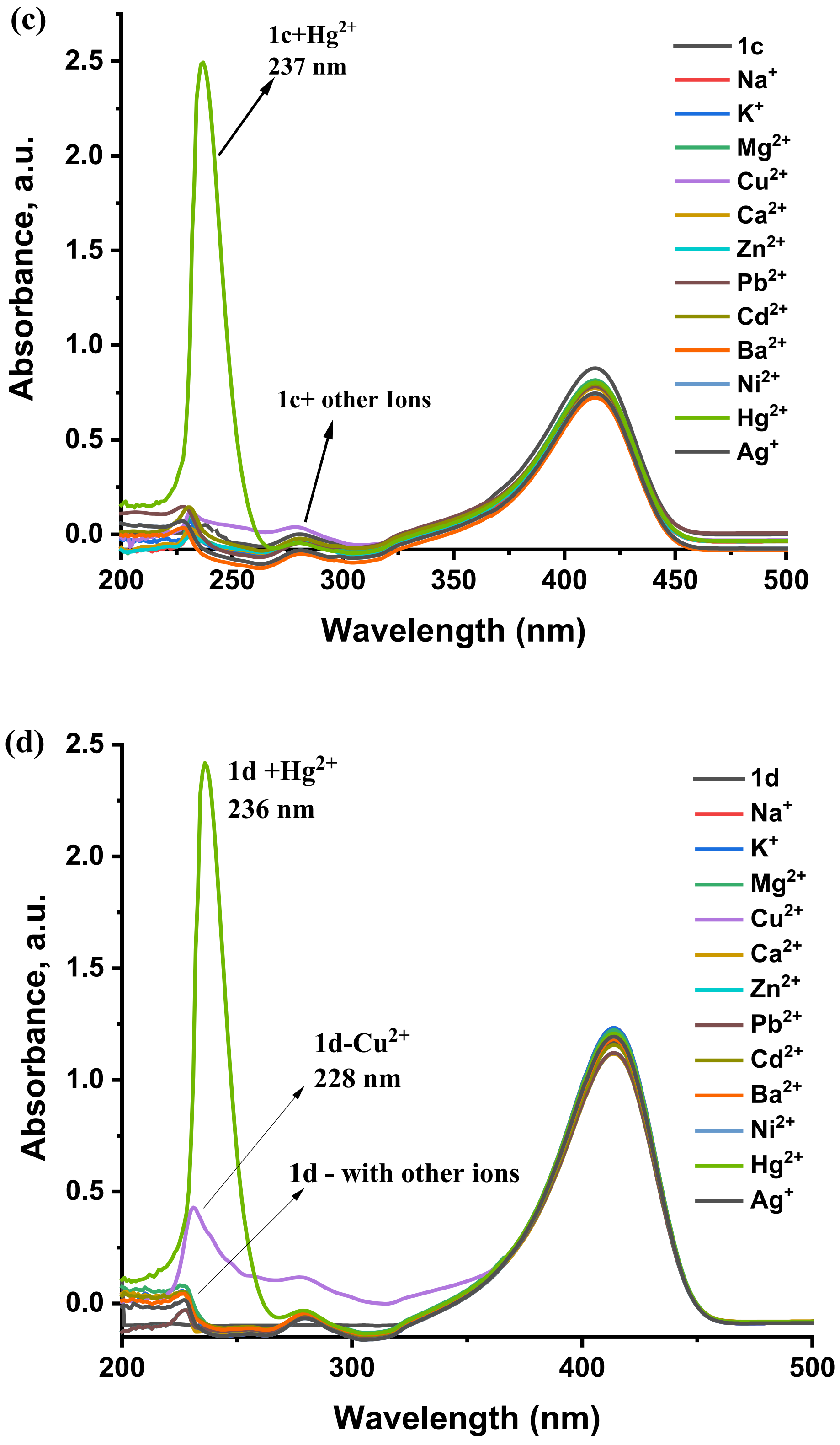

Figure S2: Absorption spectra of the **1a-1d** probe response (20 μM) to different metal ions (40 μM) in H_2O: PBS buffer solution (3:1, v/v, pH=7.4) at λ= 200-500 nm.

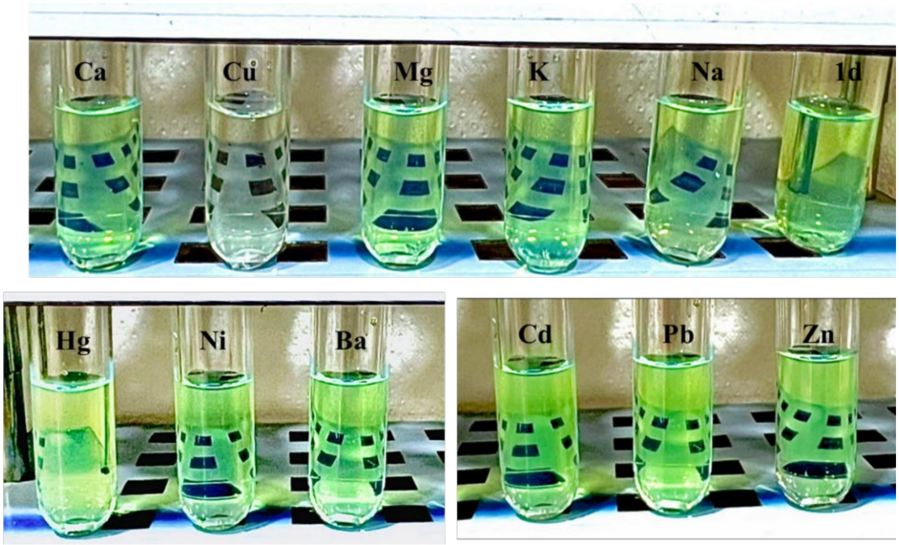

Figure S3: Photographs depicting distinct behavior of **1d** in the presence of Hg^{2+}, Cu^{2+}, and other ions.

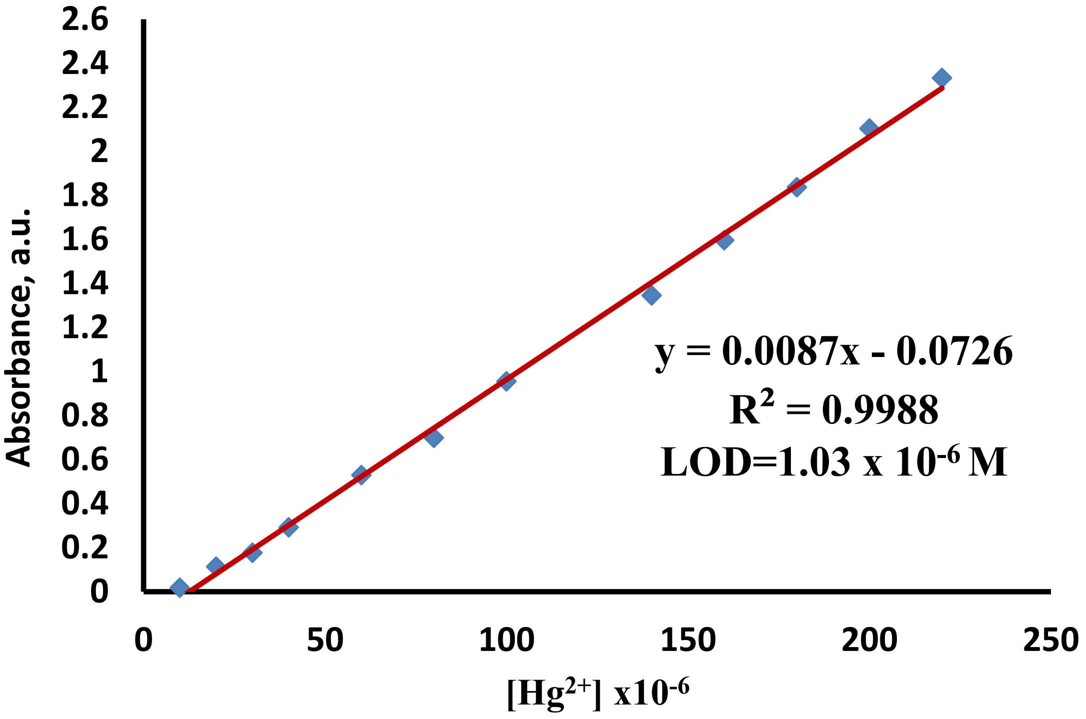

Figure S4: Fitting plot of absorption titration for Hg^{2+} ion (0-220 μM) with **1d** probe (20 μM) at λ= 236 nm.

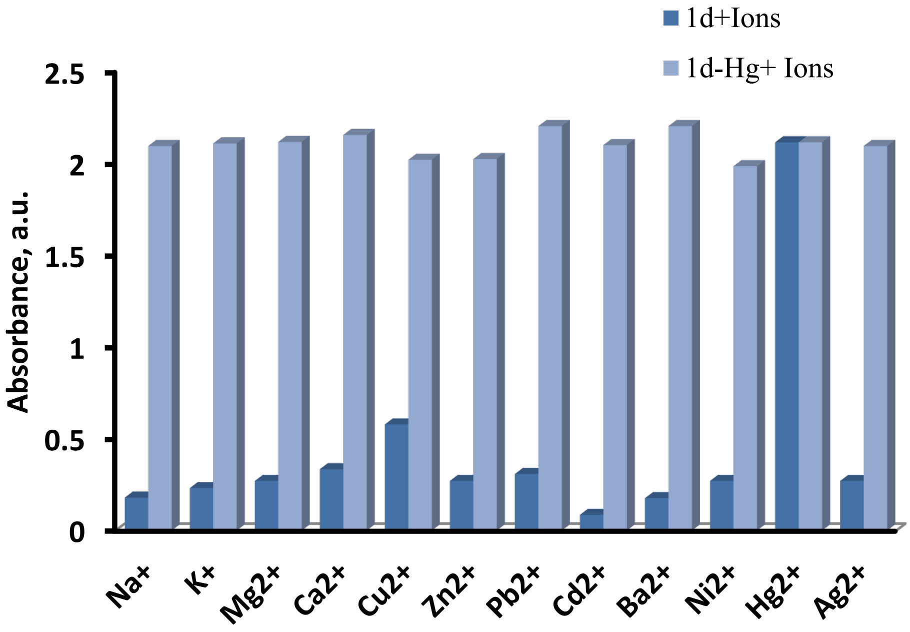

Figure S5: Selectivity of **1d** probe for Hg^{2+} in the presence of other metal cations.

Fluorescence emission properties of (1a-1d) :

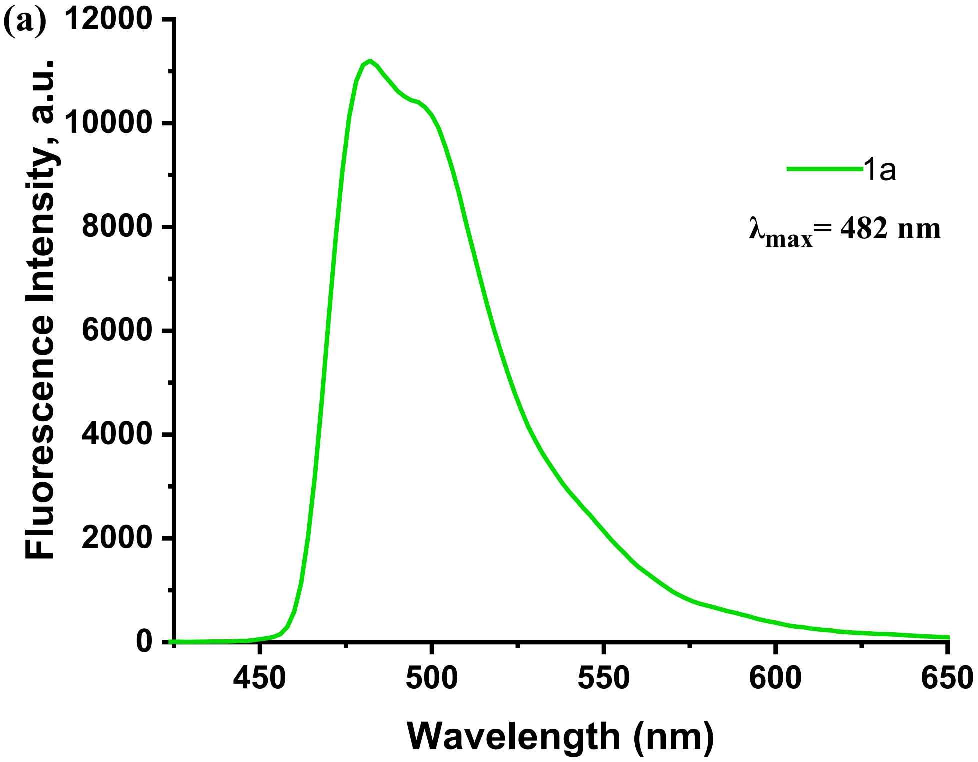

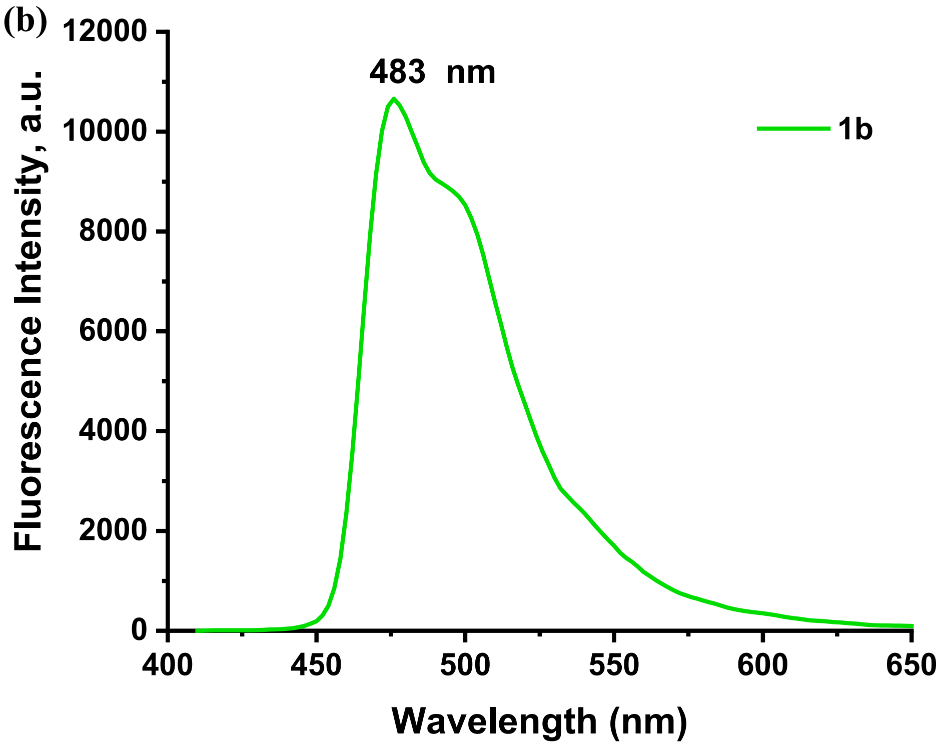
(b)
483 nm
1b
Fluorescence Intensity, a.u.
Wavelength (nm)
0
2000
4000
6000
8000
10000
12000
400
450
500
550
600
650

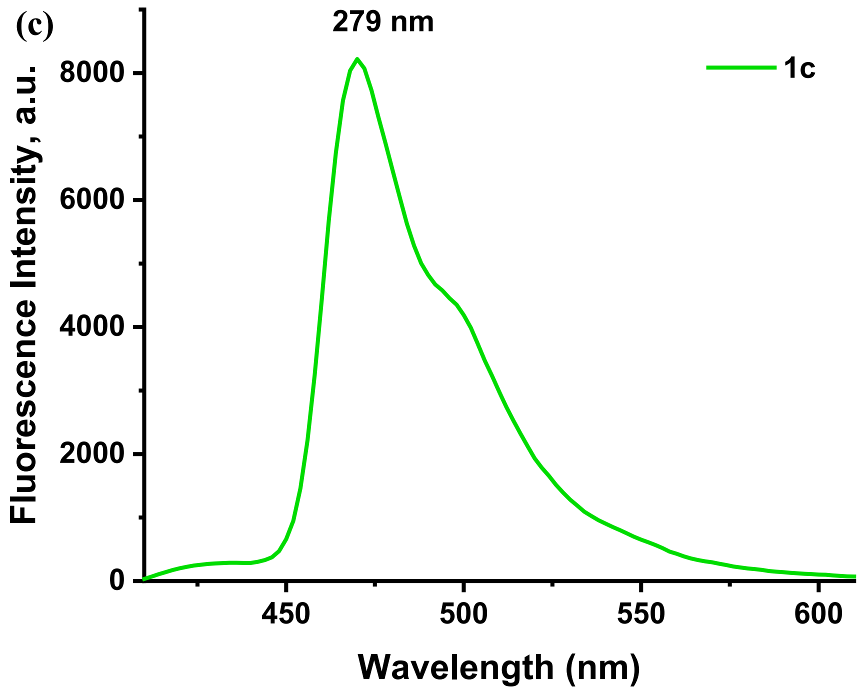
(c)
279 nm
1c
Fluorescence Intensity, a.u.
Wavelength (nm)
0
2000
4000
6000
8000
450
500
550
600

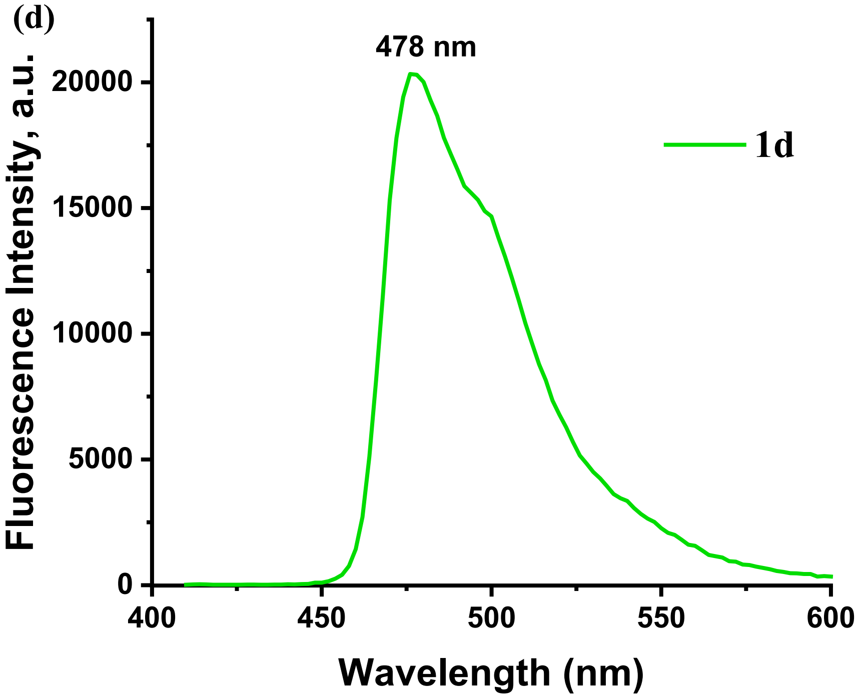

Figure S6: The fluorescence emission spectra of compounds (a) **1a** (b) **1b**, (c)**1c**, and (d) **1d** (20 μM) in solution of DMSO:H_2O (2:8 v/v, pH=7.4) at room temperature and wavelength range 400-600 nm.

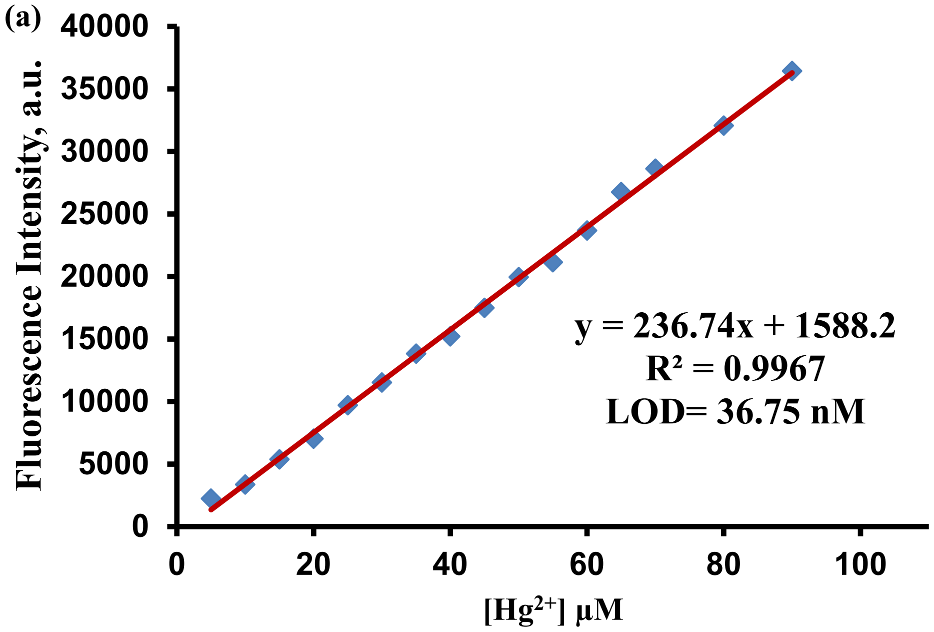

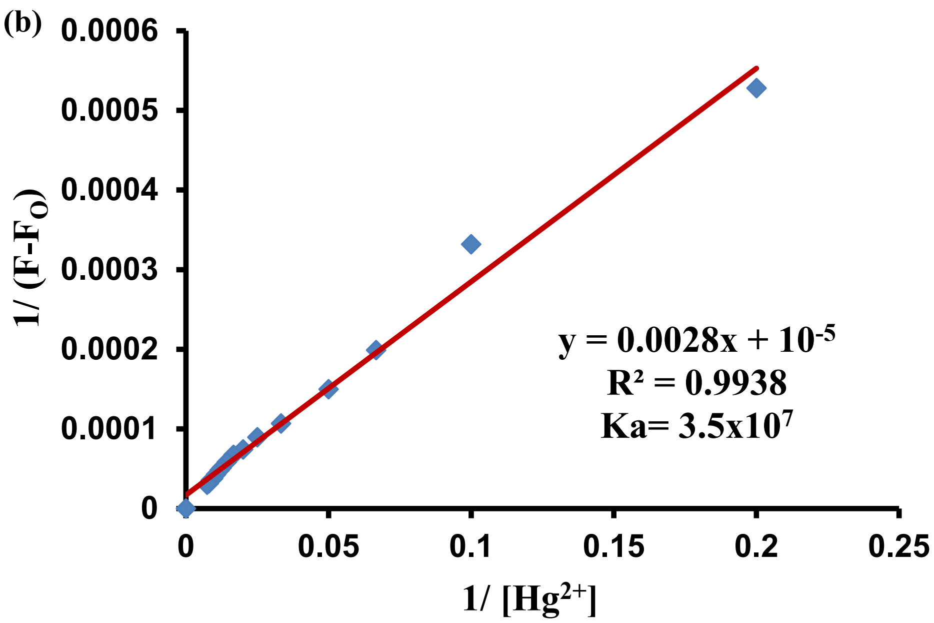

Figure S7: (a) Fitting plot of linear relationship of probe 1d with Hg^{2+} concentration; (b) Plot of (1/(F-F$_o$)) vs (1/Hg^{2+}).

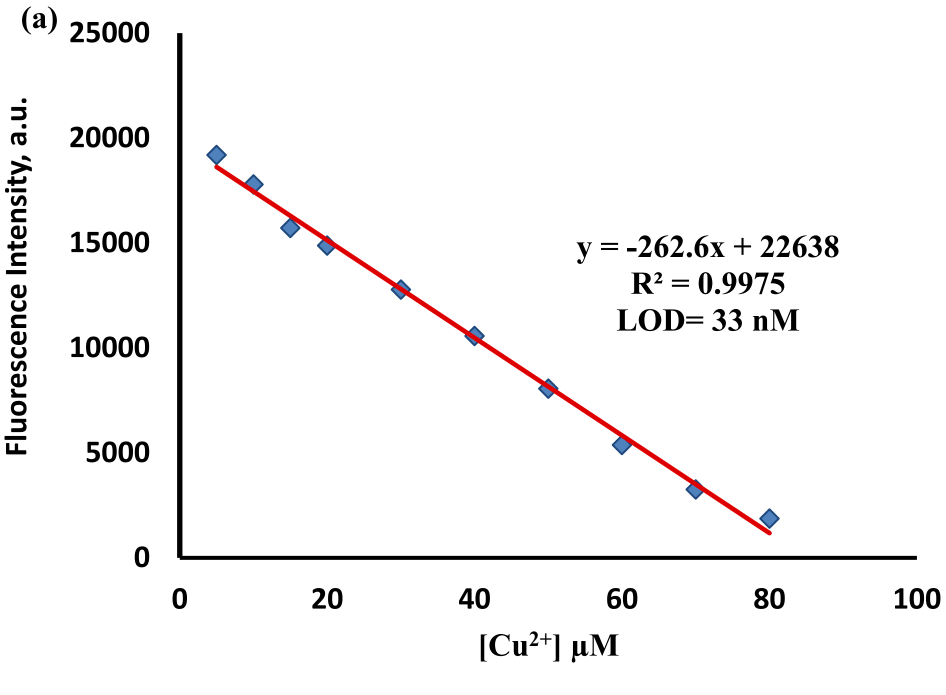

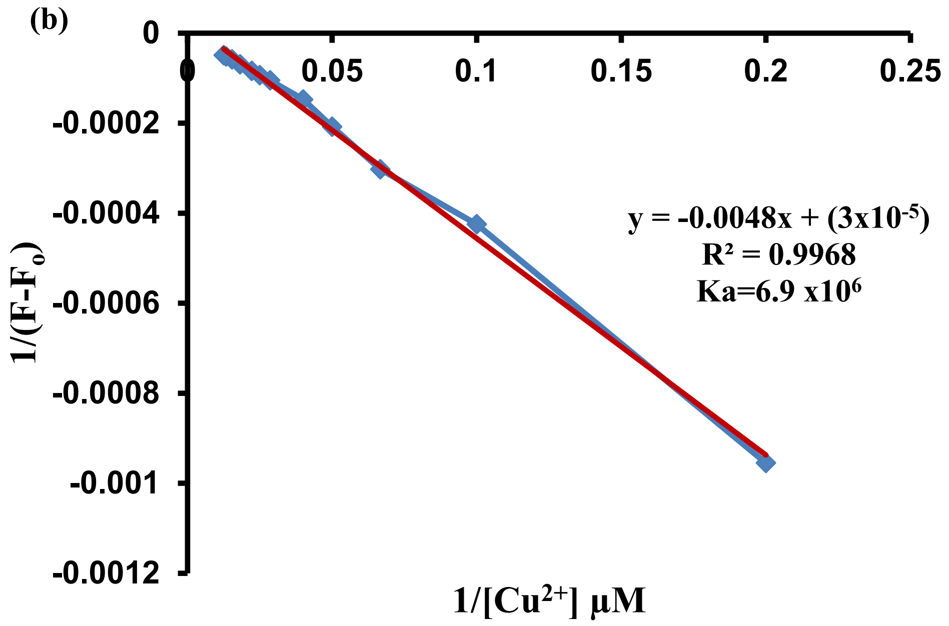

Figure S8: (a) Fitting plot of linear relationship of probe 1d with Cu^{2+} concentration; (b) Plot of (1/(F-F_o)) vs (1/Cu^{2+}).

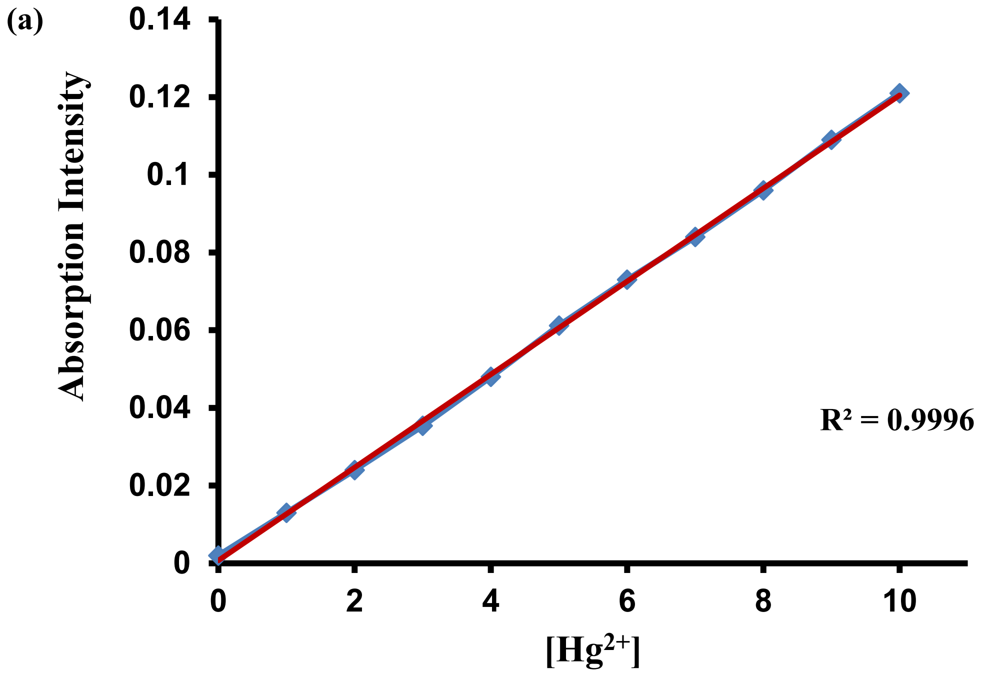

(b)

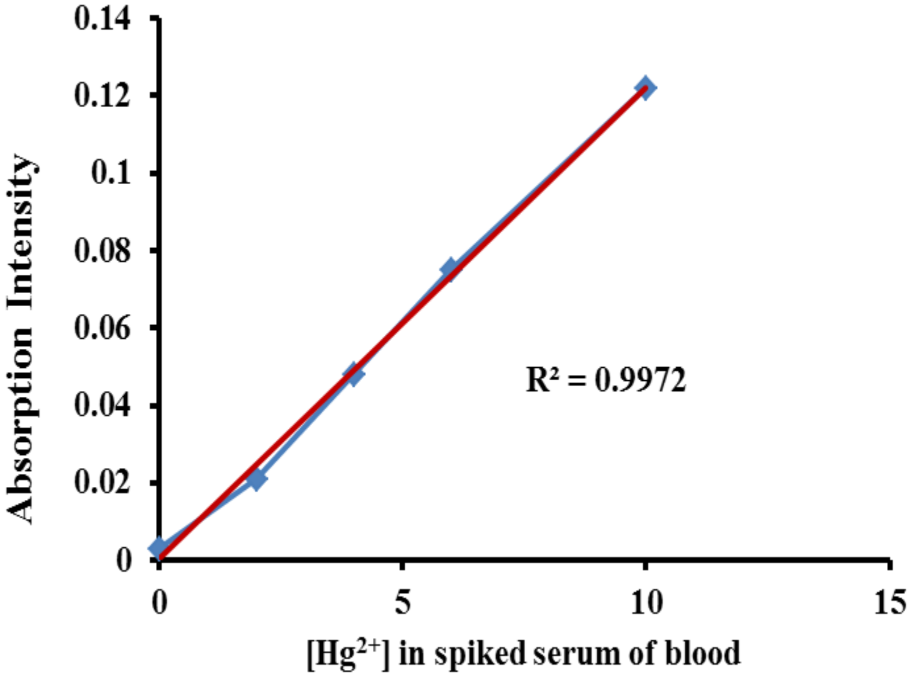

Figure S9: (a) Absorption calibration curve of **1d** (10 μM) with Hg^{2+} ion (0-10 μM), (b) Absorption intensity of **1d** (10 μM) with spiked Hg^{2+} ion (0, 2, 4, 6, 8, 10 μM) in serum blood sample.

(a)

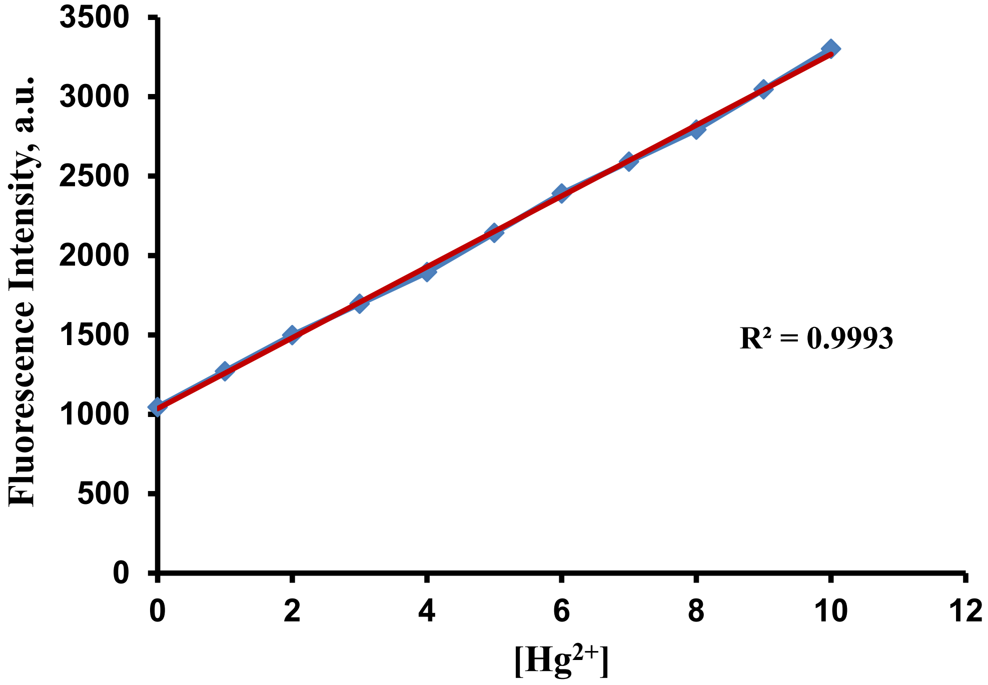

(b)

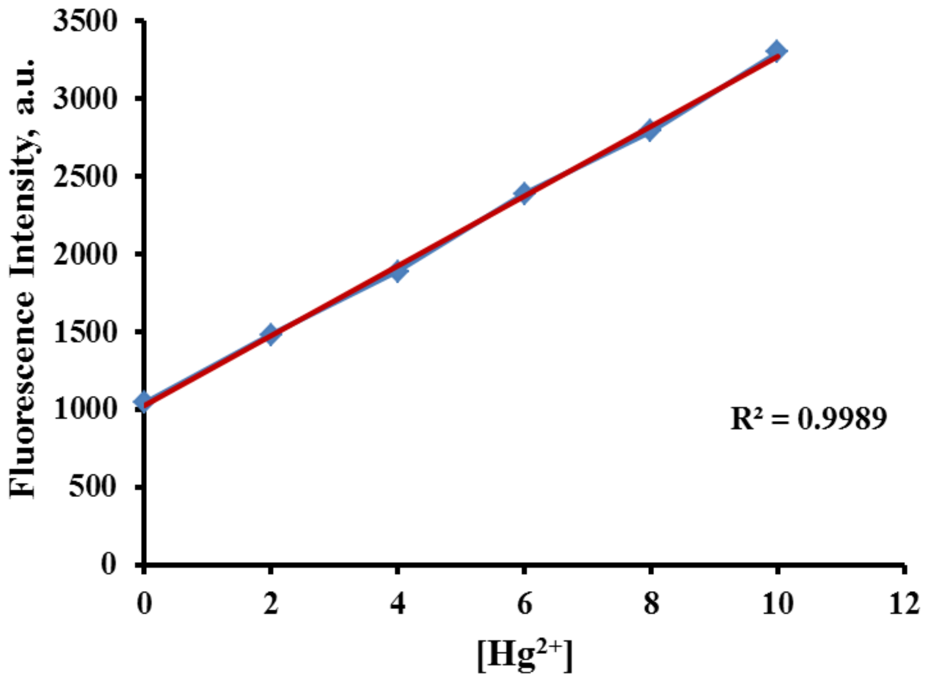

Figure S10: (a) Fluorescence calibration curve of **1d** (10 µM) with Hg^{2+} ion (0-10 µM), (b) Fluorescence intensity of **1d** (10 µM) with spiked Hg^{2+} ion (0, 2, 4, 6, 8, 10 µM) in serum blood sample .

(a)

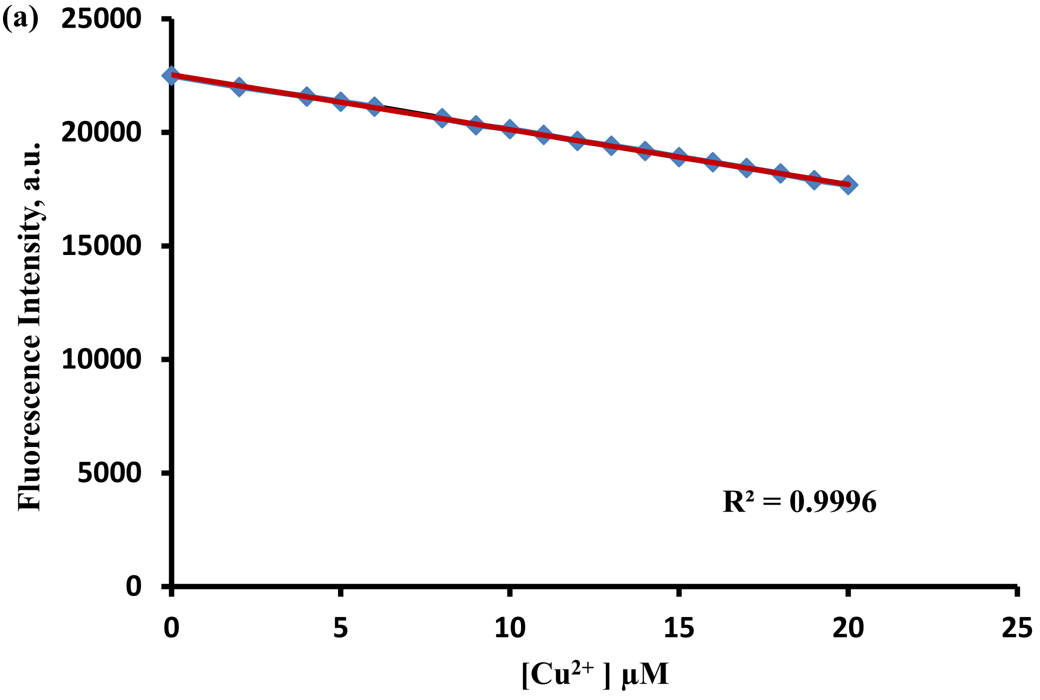

(b)

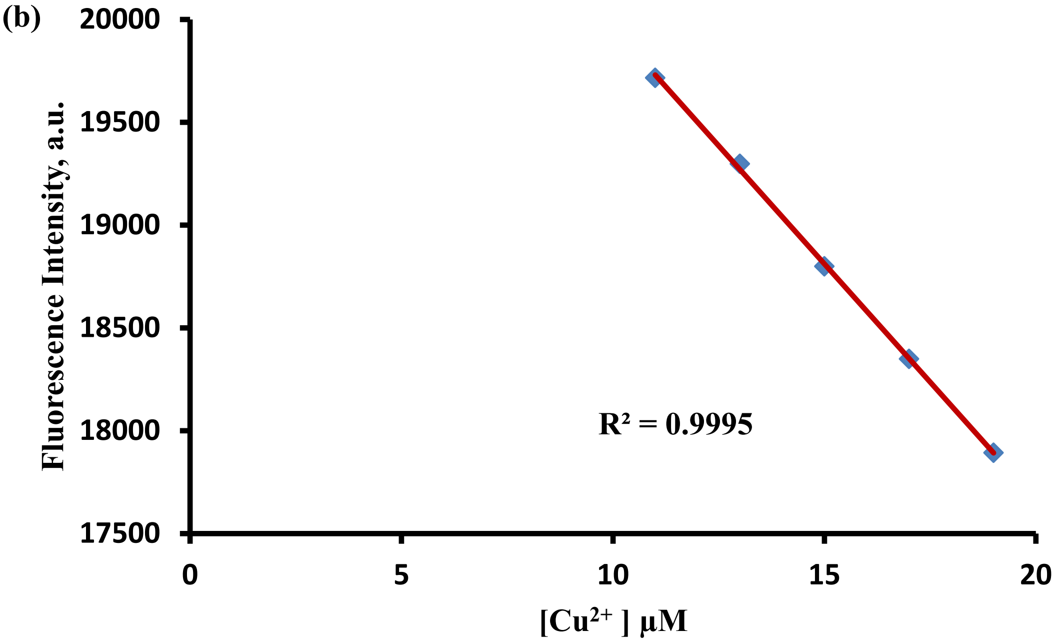

Figure S11: (a) Fluorescence calibration curve of **1d** (10 µM) with Cu^{2+} ion (0-20µM), (b) Fluorescence intensity of **1d** (10 µM) with spiked Cu^{2+} ion (0, 2, 4, 6, 8, 10 µM) in serum blood sample.

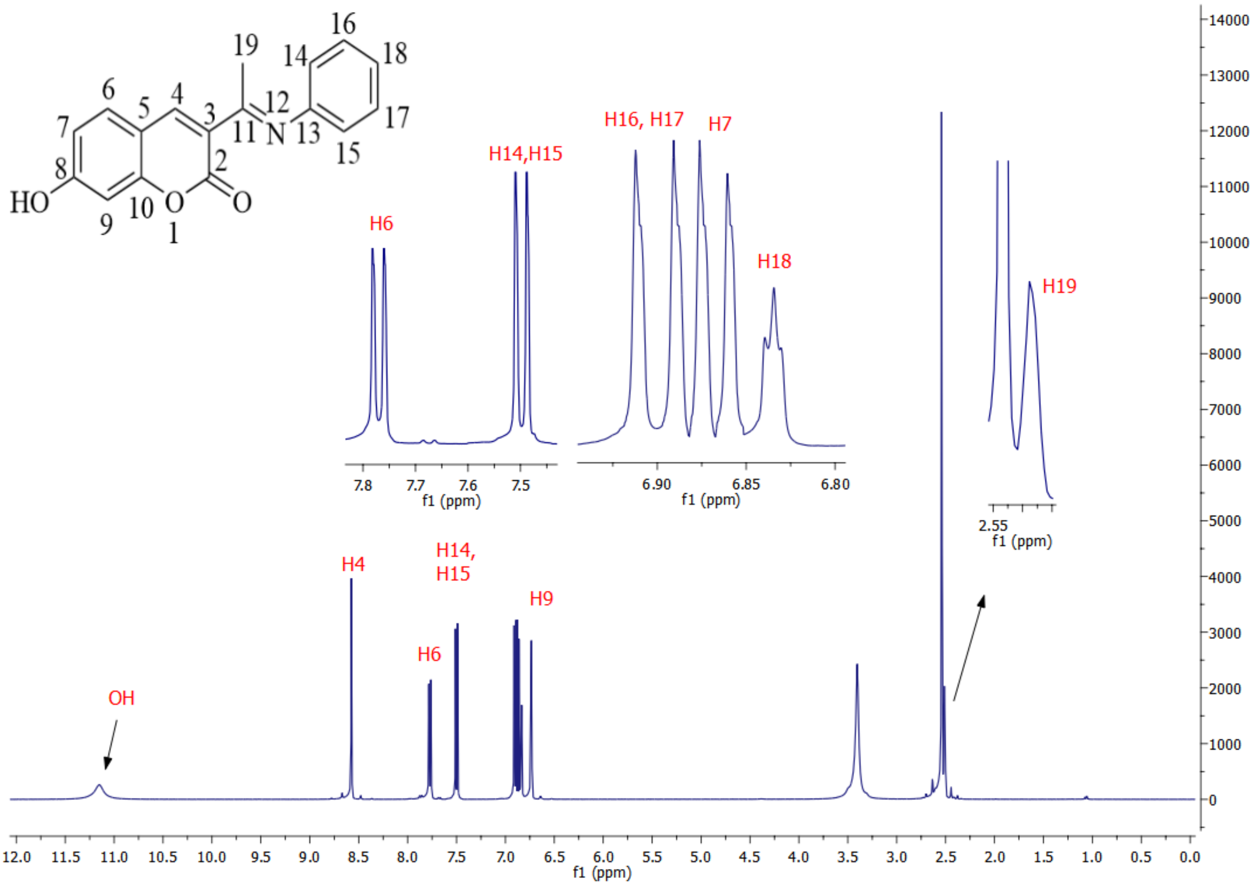

Figure S12: ^{1}H-NMR-spectrum of compound **1a**.

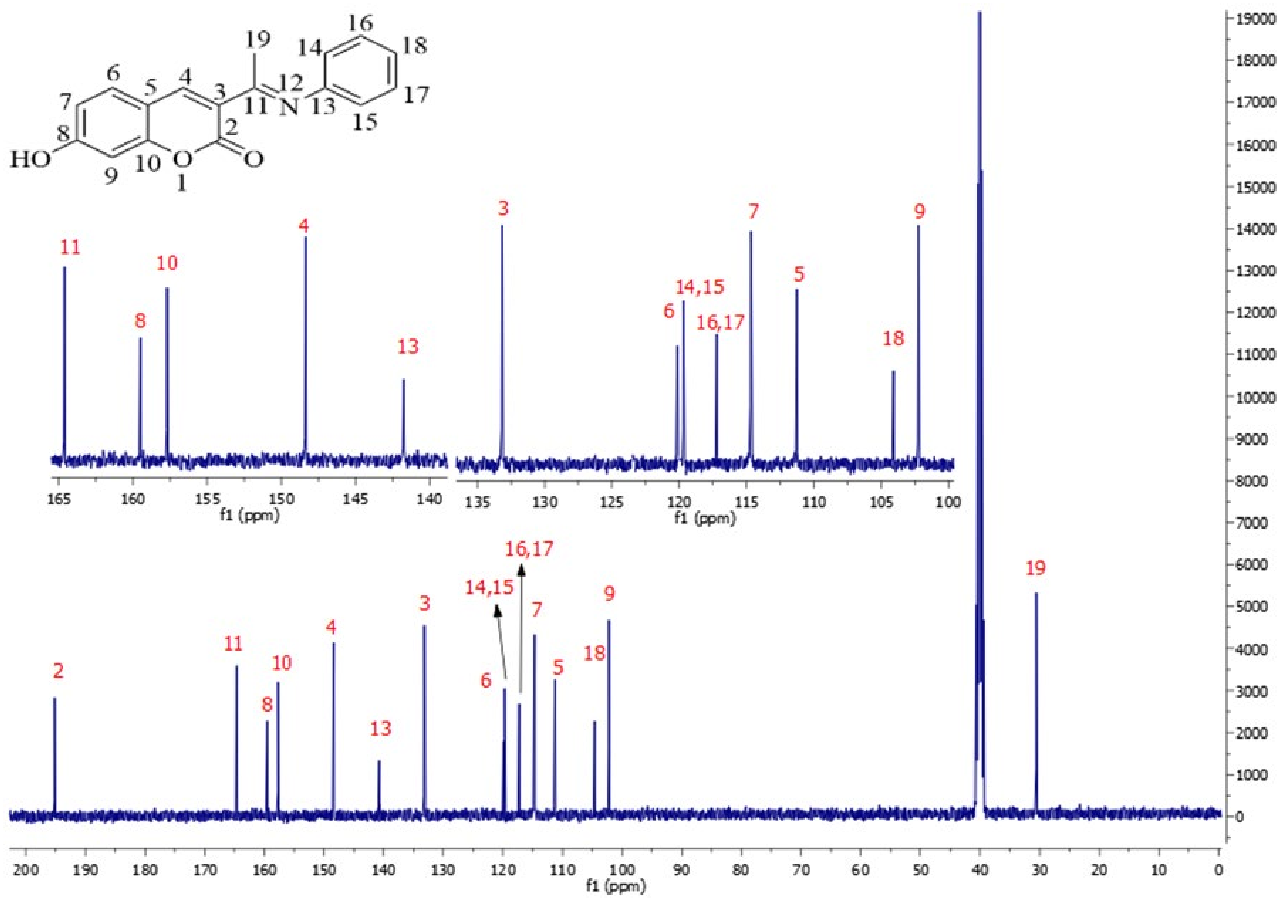

Figure S13: ^{13}C-NMR-spectrum of compound **1a**.

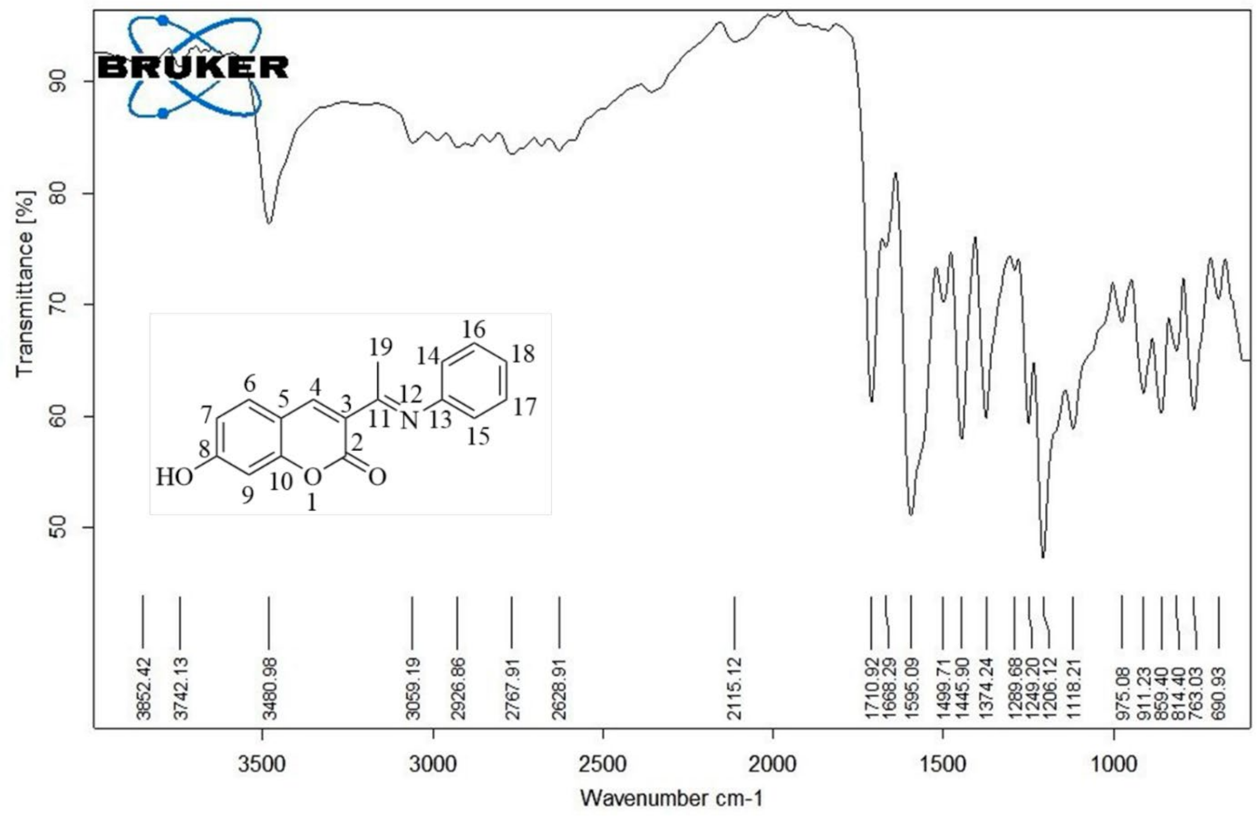

Figure S14: FT-IR spectrum of compound **1a**.

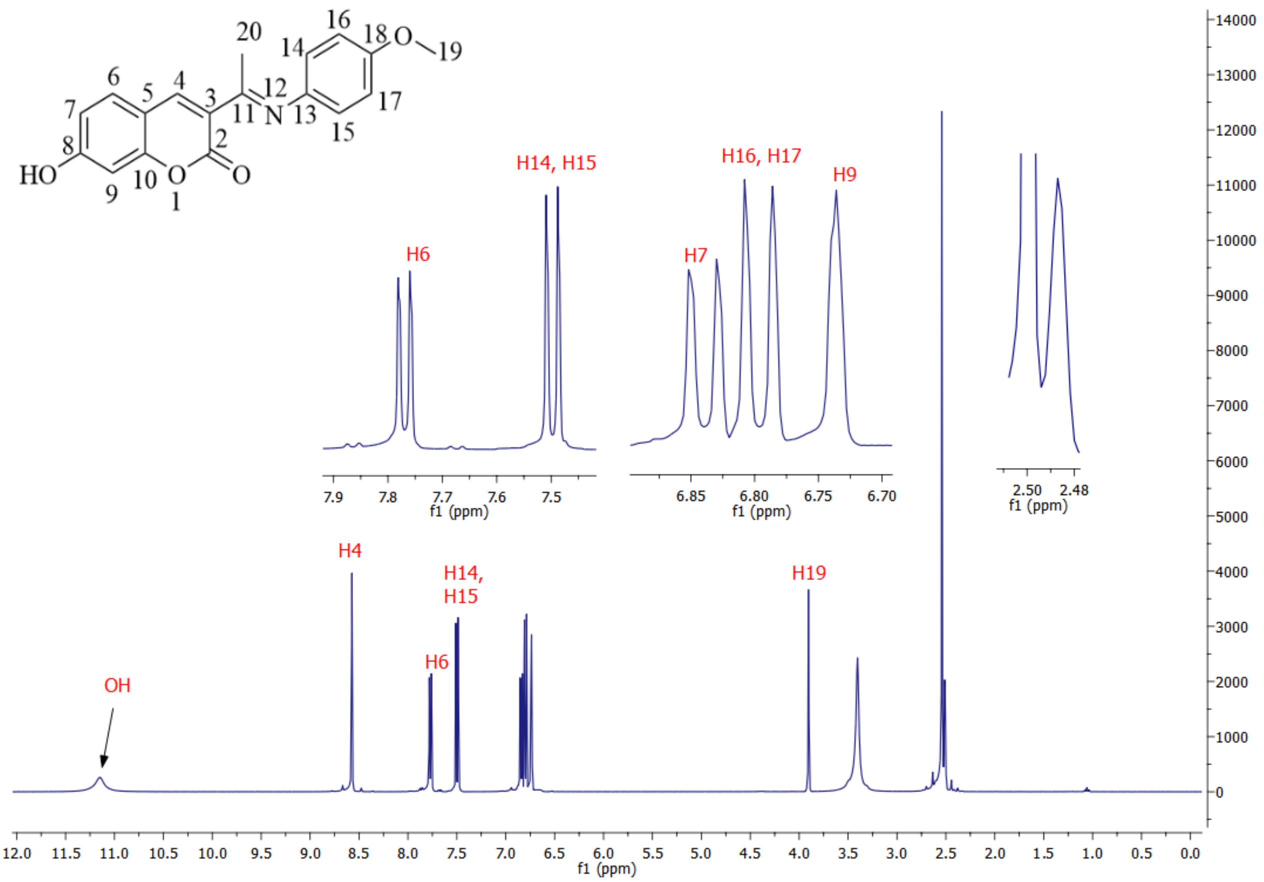

Figure S15: ^{1}H-NMR-spectrum of compound **1b**.

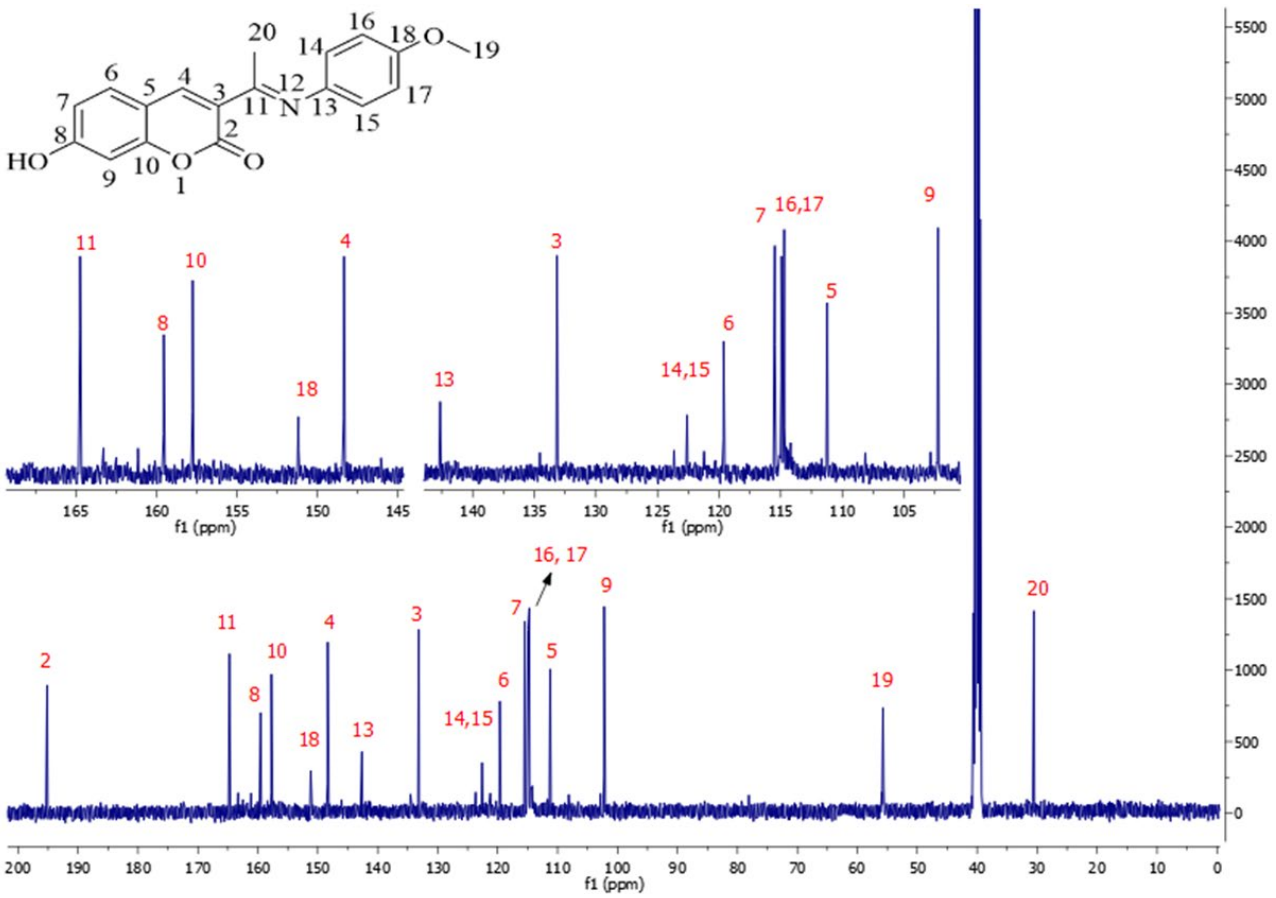

Figure S16: ^{13}C-NMR-spectrum of compound **1b**.

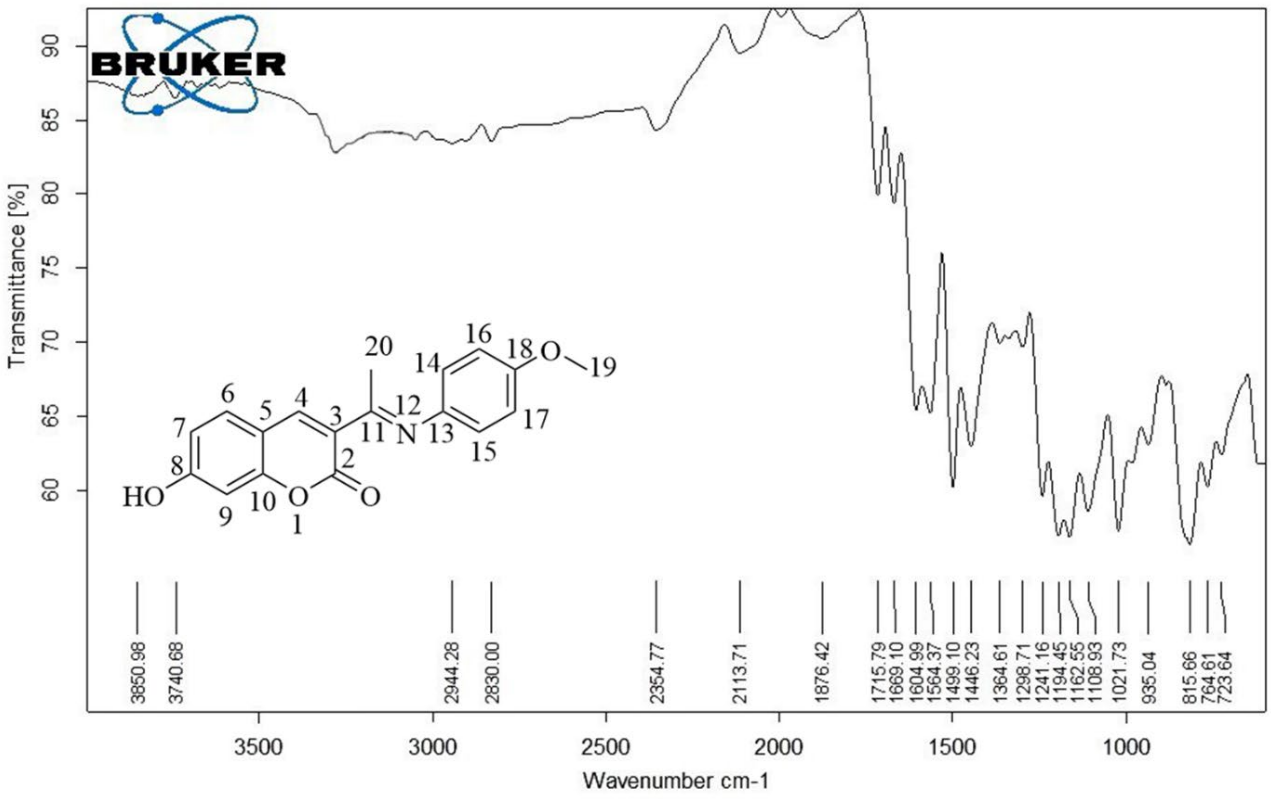

Figure S17: FT-IR spectrum of compound **1b**.

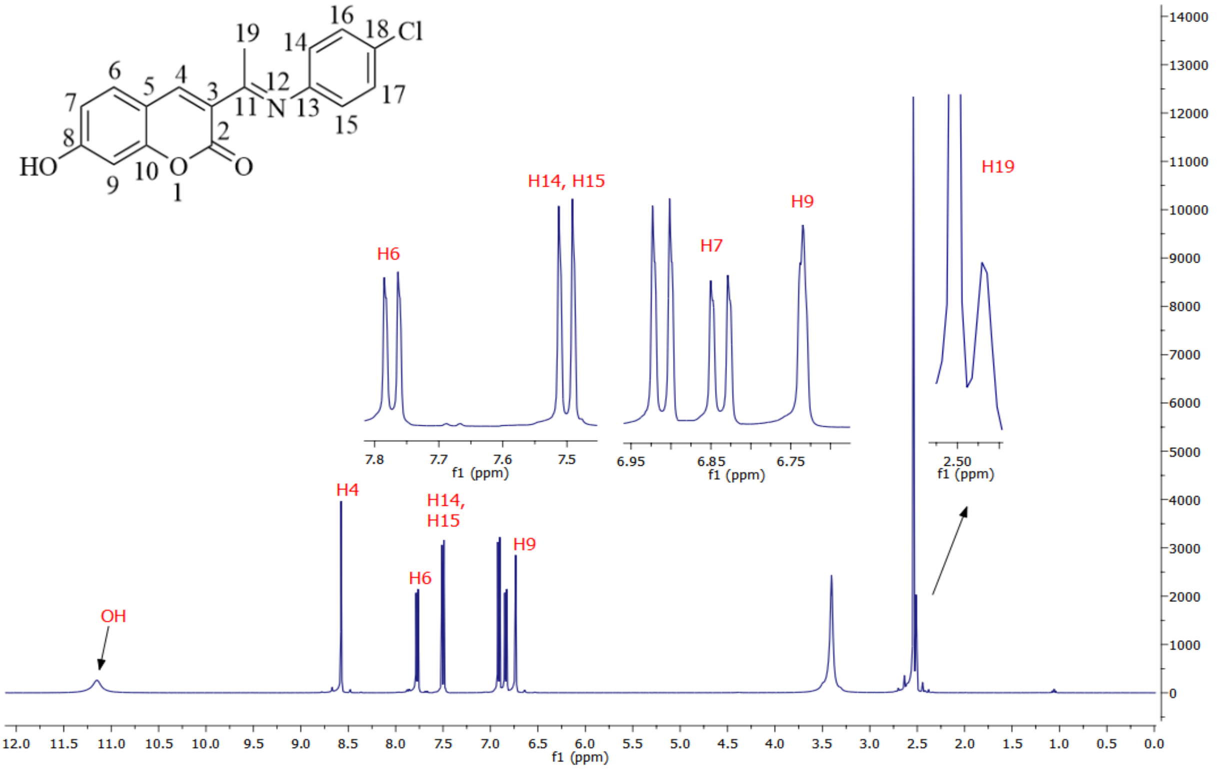

Figure S18: ^{1}H-NMR-spectrum of compound **1c**.

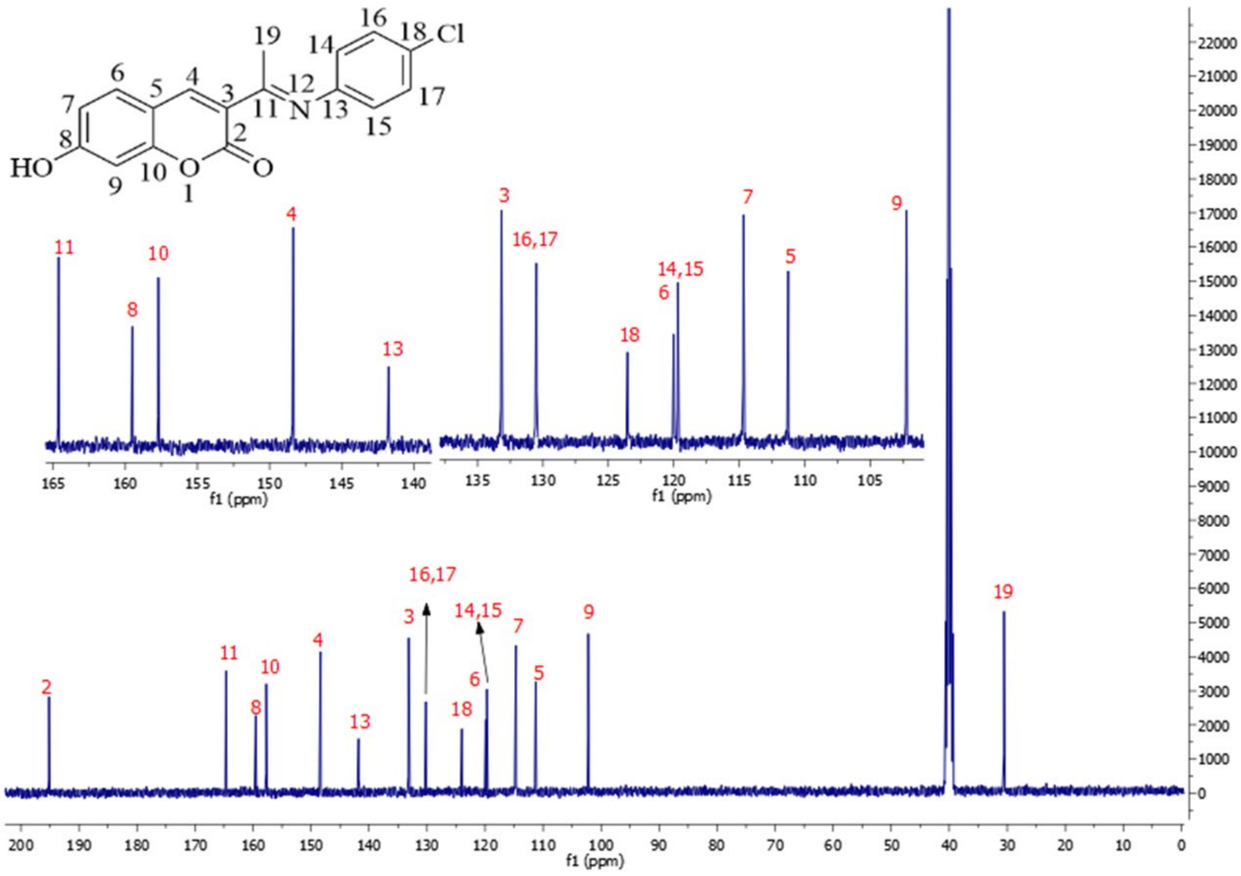

Figure S19: ^{13}C-NMR-spectrum of compound **1c**.

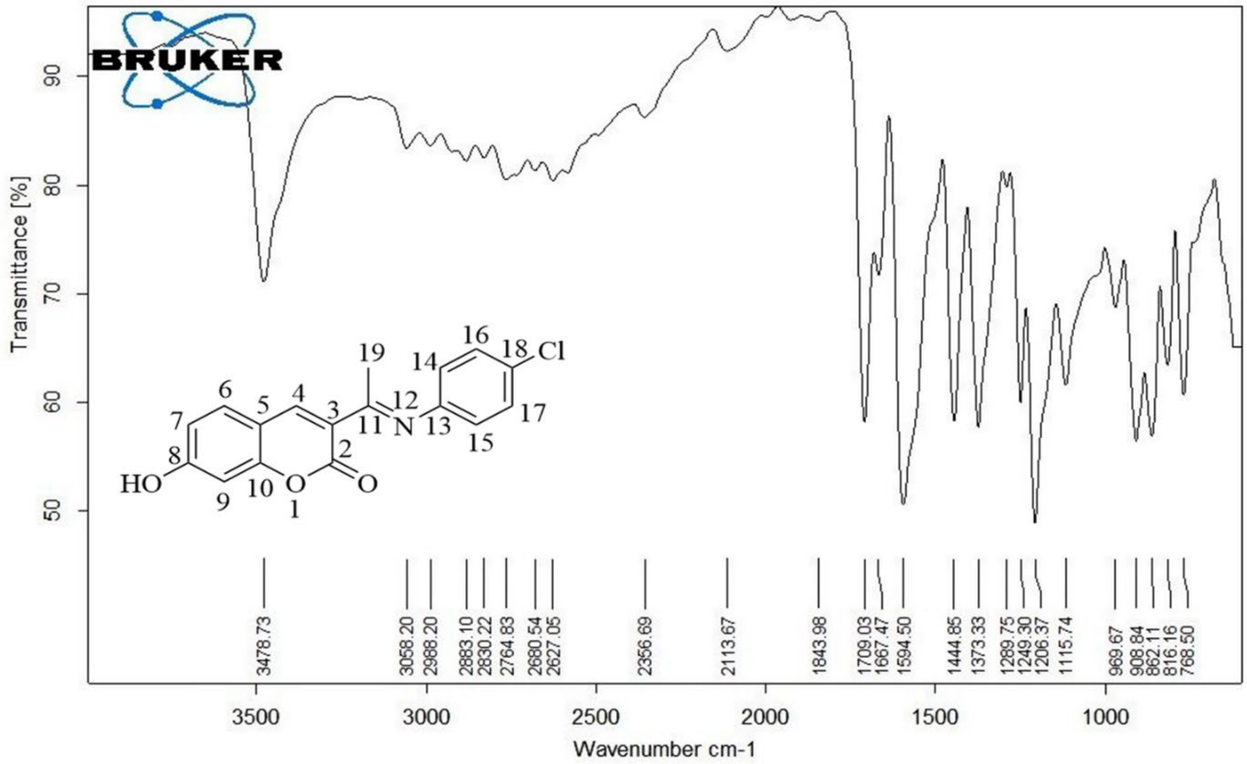

Figure S20: FT-IR spectrum of compound **1c**.

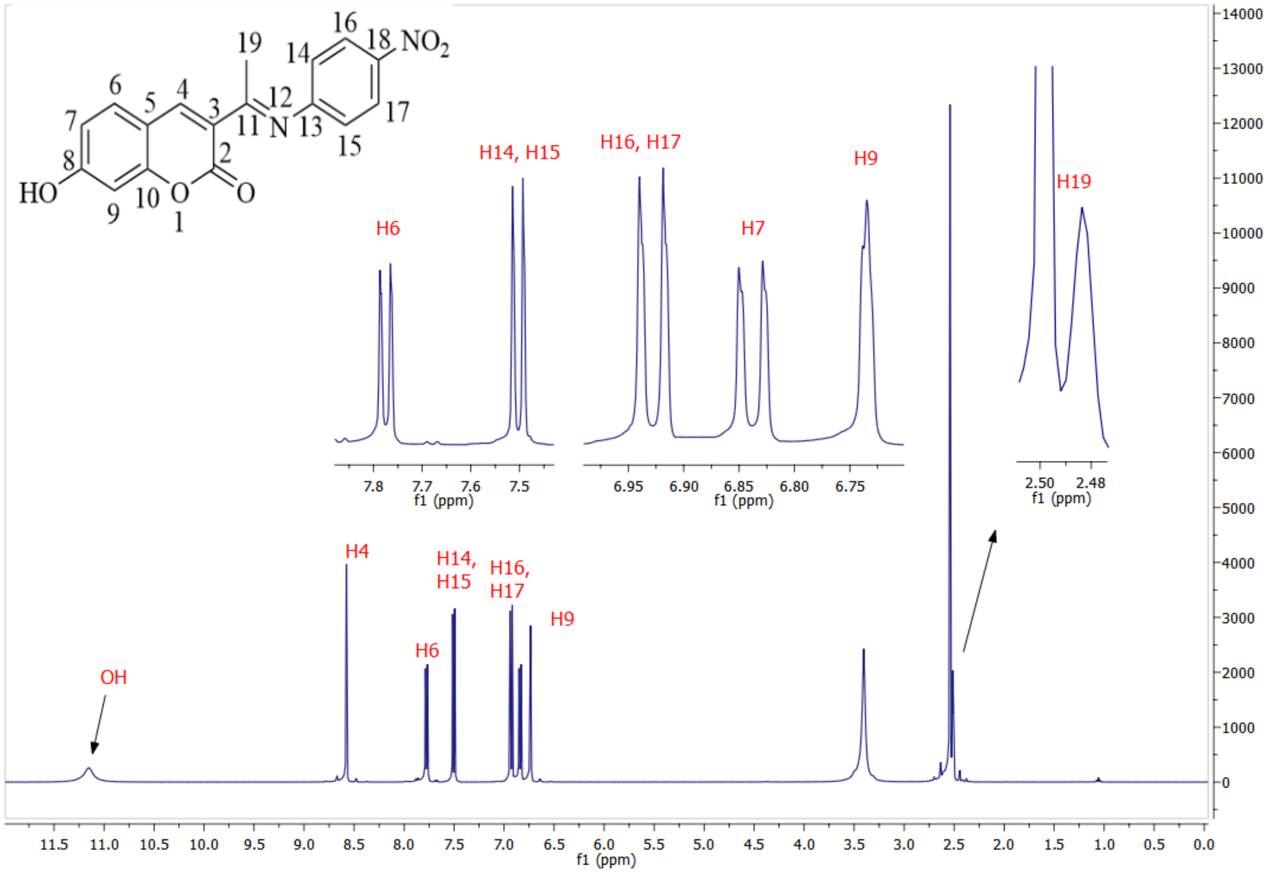

Figure S21: ^{1}H-NMR-spectrum of compound **1d**.

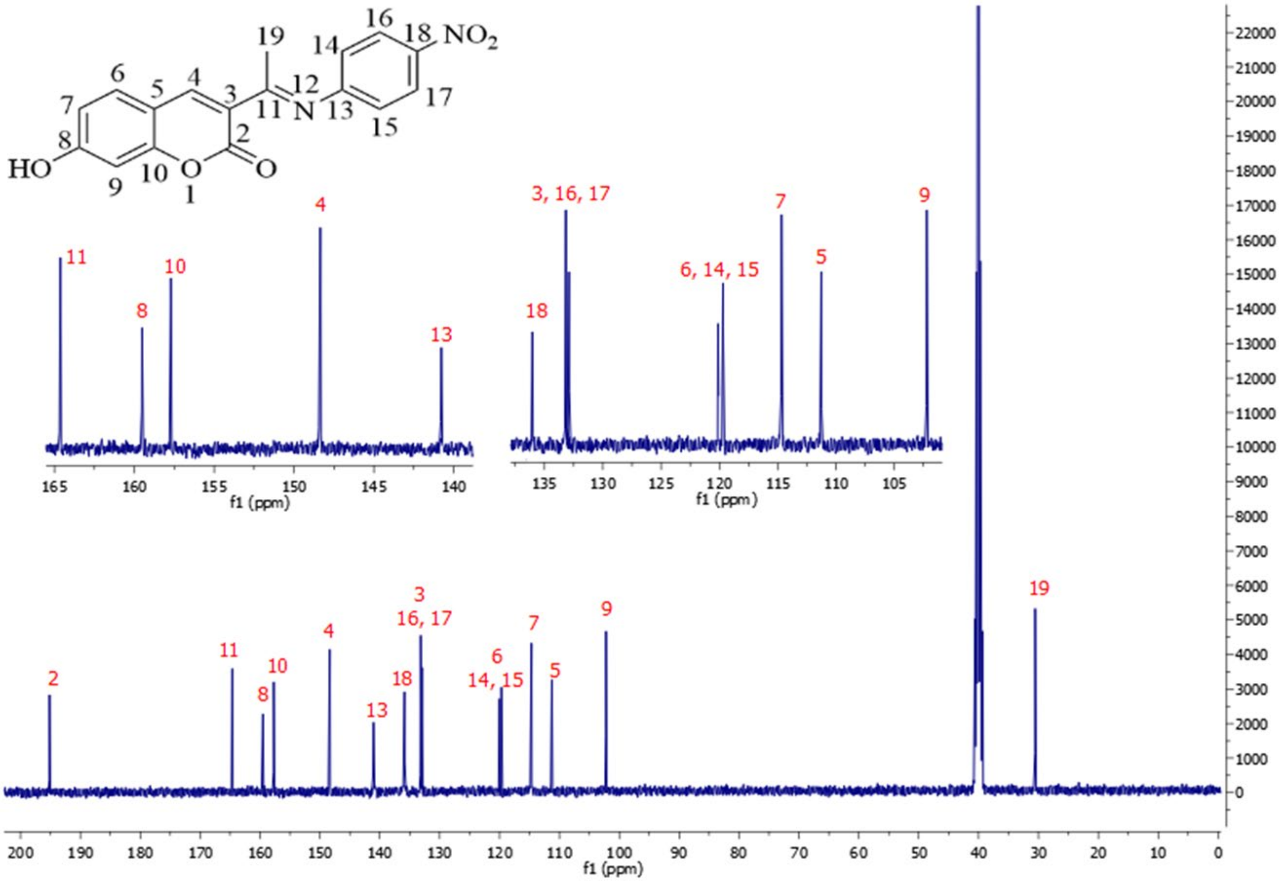

Figure S22: ^{13}C-NMR-spectrum of compound **1d**.

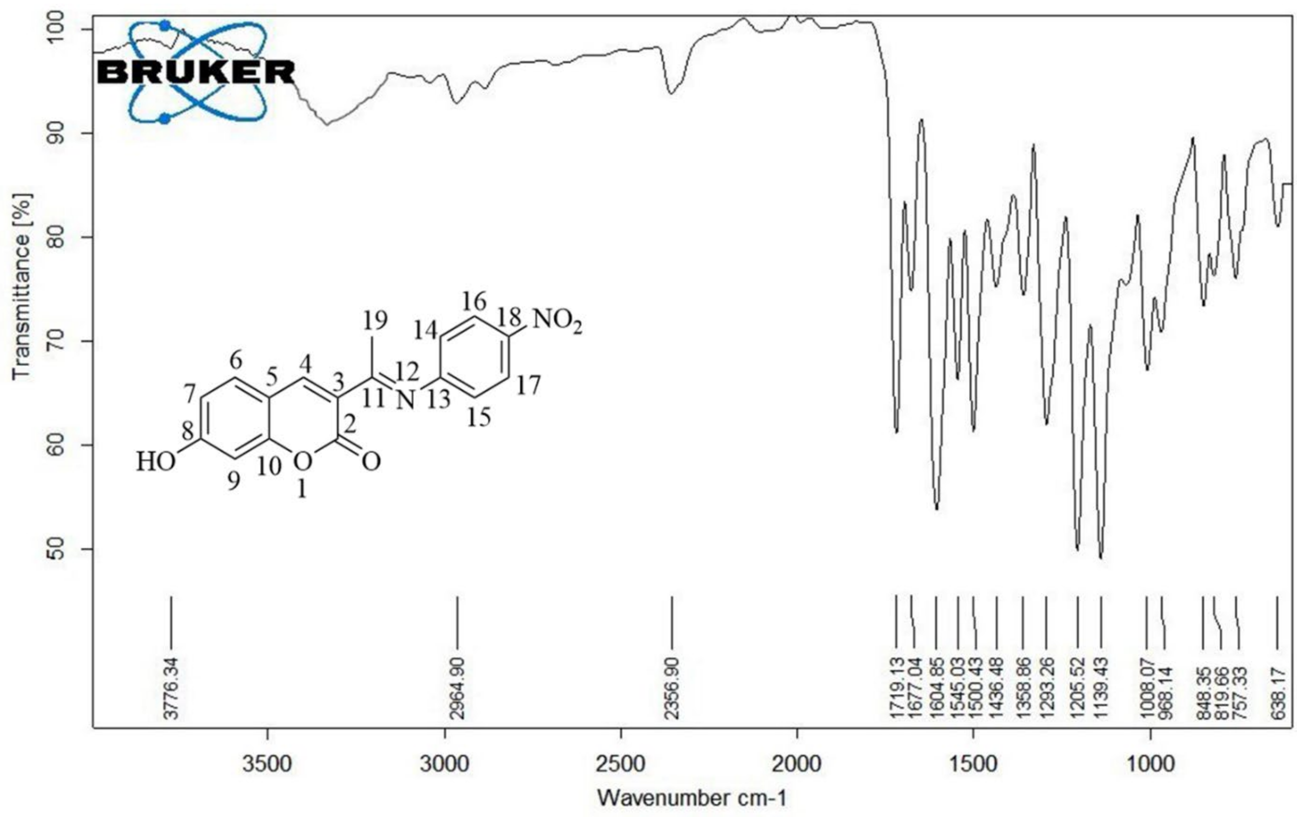

Figure S23: FT-IR spectrum of compound **1d**.

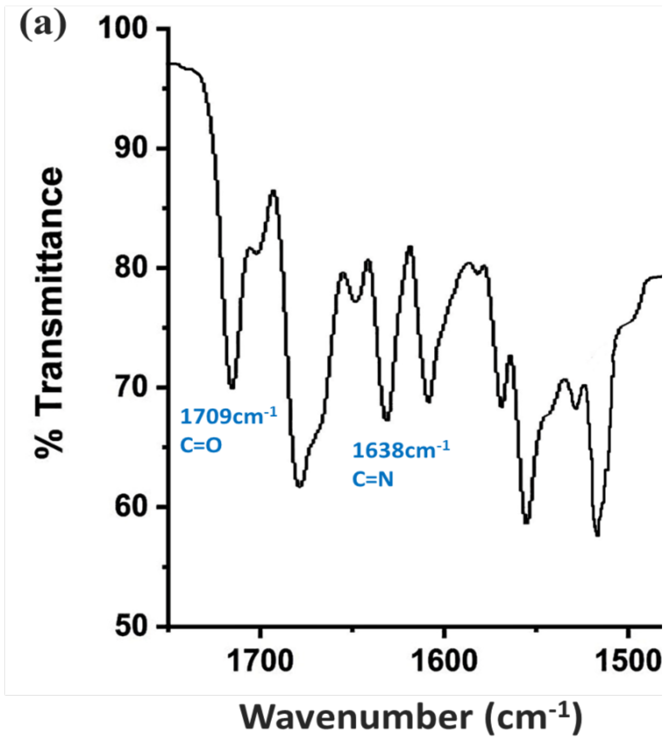

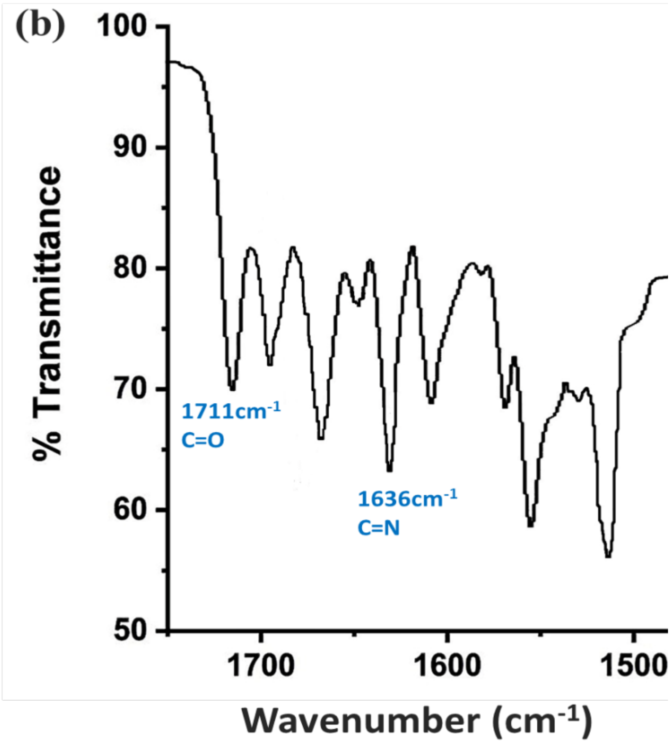

Figure S24: FT-IR spectrum of (a) probe 1d-Hg^{2+}, (b) 1d-Cu^{2+}.
